# Regulated cell death and DAMPs as biomarkers and therapeutic targets in normothermic perfusion of transplant organs. Part 1: their emergence from injuries to the donor organ

**DOI:** 10.3389/frtra.2025.1571516

**Published:** 2025-04-24

**Authors:** Walter G. Land, Andreas Linkermann

**Affiliations:** ^1^ German Academy for Transplantation Medicine, Munich, Germany; ^2^ Laboratoire d'ImmunoRhumatologie Moléculaire, plateforme GENOMAX, INSERM UMR_S 1109, Faculté de Médecine, Fédération Hospitalo-Universitaire OMICARE, Fédération de Médecine Translationnelle de Strasbourg (FMTS), Institut Thématique Interdisciplinaire TRANSPLANTEX NG, Université de Strasbourg, Strasbourg, France; ^3^ Department of Integrated Medical Sciences, Medical Science Faculty, State University of Rio De Janeiro, Cabo Frio, Brazil; ^4^ Department of Medicine V, University Medical Centre Mannheim, University of Heidelberg, Mannheim, Germany; ^5^ Division of Nephrology, Department of Internal Medicine 3, University Hospital Carl Gustav Carus at the Technische Universität Dresden, Dresden, Germany; ^6^ Division of Nephrology, Department of Medicine, Albert Einstein College of Medicine, Bronx, NY, United States

**Keywords:** innate alloimmunity, injuries to donor organs, DAMPs, regulated cell death, normothermic regional perfusion, normothermic machine perfusion, donation after brain death, donation after circulatory death

## Abstract

This Part 1 of a bipartite review commences with a succinct exposition of innate alloimmunity in light of the danger/injury hypothesis in Immunology. The model posits that an alloimmune response, along with the presentation of alloantigens, is driven by DAMPs released from various forms of regulated cell death (RCD) induced by any severe injury to the donor or the donor organ, respectively. To provide a strong foundation for this review, which examines RCD and DAMPs as biomarkers and therapeutic targets in normothermic regional perfusion (NRP) and normothermic machine perfusion (NMP) to improve outcomes in organ transplantation, key insights are presented on the nature, classification, and functions of DAMPs, as well as the signaling mechanisms of RCD pathways, including ferroptosis, necroptosis, pyroptosis, and NETosis. Subsequently, a comprehensive discussion is provided on major periods of injuries to the donor or donor organs that are associated with the induction of RCD and DAMPs and precede the onset of the innate alloimmune response in recipients. These periods of injury to donor organs include conditions associated with donation after brain death (DBD) and donation after circulatory death (DCD). Particular emphasis in this discussion is placed on the different origins of RCD-associated DAMPs in DBD and DCD and the different routes they use within the circulatory system to reach potential allografts. The review ends by addressing another particularly critical period of injury to donor organs: their postischemic reperfusion following implantation into the recipient—a decisive factor in determining transplantation outcome. Here, the discussion focuses on mechanisms of ischemia-induced oxidative injury that causes RCD and generates DAMPs, which initiate a robust innate alloimmune response.

## 1 Prologue

### 1.1 Allograft injury-induced innate alloimmunity

**Figure 1:**
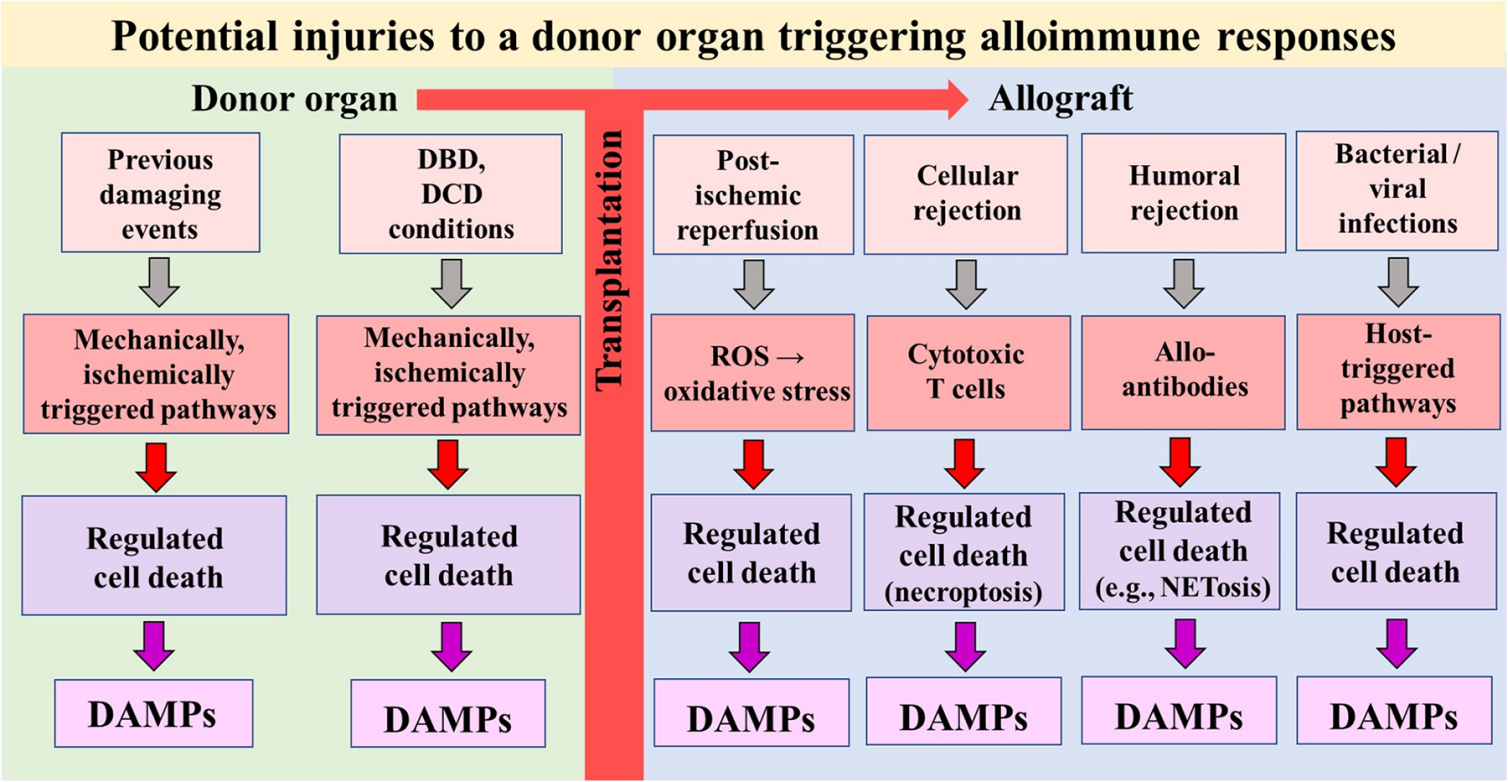
Simplified schematic diagram of a model illustrating potential injuries that can affect a donor organ before and after transplantation into a recipient. The injuries can induce types of regulated cell death that serve as sources of DAMPs. In turn, DAMPs—via activation of pattern recognition receptor-bearing cells of the donor's and recipient's innate immune system—drive alloimmune responses (not shown). DBD, donation after brain death; DCD, donation after circulatory death; ROS, reactive oxygen species.

The 23-year-old conceptual model of allograft injury [in particular, ischemia/reperfusion injury (IRI)] as the primary spark that initiates an innate immune response and subsequently promotes a specific adaptive alloimmune response resulting in allograft rejection (“Innate Alloimmunity”) is gradually coming of age (1–3). Rooted in the Danger/Injury Hypothesis in Immunology and launched 31 years ago (4, 5), the concept understands allograft injury—in tandem with the presence of donor alloantigens—as the primary key trigger for eliciting innate alloimmune responses. The core of the Danger/Injury Model is reflected in its postulate that *pattern recognition receptor* (PRR)-bearing cells of the innate immune system perceive any cell stress and any tissue damage via recognition of stress and damage-generated *damage-associated molecular patterns* (“DAMPs”), a term coined in 2003 (6), and also referred to as “danger signals” or “alarmins” (7) in the international literature. A seminal breakthrough in the study of injury-promoted DAMP emission emerged with the recognition that these distinct molecules are released from cells undergoing stress-/injury-induced regulated cell death (RCD) (8). Accordingly, it is increasingly accepted in modern transplantology research that any severe damage to the donor organ before and after transplantation—including T cell- mediated- (9, 10) and alloantibody-mediated allograft rejection episodes (11)—triggers types of RCD, which may serve as a prolific sources of extracellularly released DAMPs (Figure 1). In other words: the intragaft identification of RCD types and associated release of DAMPs, which indicate the level of injury to a donor organ, provide the involved transplant surgeon with two essential pieces of information: an assessment of the degree of (i) viability of the organ and (ii) its immunogenicity, that is, its capacity to promote an innate alloimmune response in the recipient. And it is this second insight that opens a window of opportunity to suppress innate alloimmune responses in an unimaginable way: namely by therapeutically targeting allograft injury-induced RCD and DAMPs (12).

### 1.2 Normothermic perfusion preservation techniques

Coincidentally, alongside the concept of injury-induced alloimmune responses, another significant topic related to organ transplantation has emerged in recent years that allows improved assessment of donor organ quality and offers a previously unimagined opportunity for therapeutic interventions: the advancements in normothermic preservation procedures (13). Indeed, constant efforts to improve the results in organ transplantation have led to increasing attention on new modalities in organ preservation, especially, recently, on the use of *in situ* normothermic regional perfusion (NRP) (14–18) and ex situ normothermic machine perfusion (NMP) (19–25). A major advantage of these new normothermic perfusion devices is that they not only provide an opportunity to assess donor organ quality more precisely but also serve as an optimal platform for therapeutic interventions.

### 1.3 Object and scope of the review

Since this review covers these two emerging topics, it has been split into two parts. Indeed, this remarkable coincidence of two independently developed momenta in organ transplantation to improve the assessment of the quality of donor organs and to offer therapeutic interventions to enhance allograft survival virtually compels researchers to examine the possible integrative combination of these two advances in transplantology in greater detail. This endeavour is referred to in the title of the review.

To emphasize the premise of this venture, Part 1 is focused on RCD-released DAMPs that accumulate in organs from *donation after brain death* (DBD) donors in comparison to marginal organs from *donation after circulatory death* (DCDs) donors [and expanded criteria donors (ECDs)] during various periods of potential allograft injury prior to onset of the innate alloimmune response in the recipient. Building on this background,—and to aid understanding of the topics in relation to the use of RCD and DAMPs as biomarkers and therapeutic targets in NRP and NMP-, Part 2 commences with the delineation of a conceptual model outlining DAMP-driven cellular and molecular trajectories involved in injury-induced, innate alloimmune-mediated acute allograft rejection. Based on this scenario and supported by encouraging findings from the literature, proposals are then made for the use of RCD and DAMPs as biomarkers during NRP and NMP to optimize the assessment of donor organ quality. However, the key focus of our thoughts presented in Part 2 of this review, is on the ambitious and visionary goal of leveraging forms of RCD and DAMPs as therapeutic targets in NRP and NMP, with the aim of alleviating early allograft inflammation and profoundly suppressing innate alloimmune-mediated allograft rejection. This strategic approach seeks to prevent the activation of intragraft donor- and recipient-derived immature dendritic cells (iDCs) into mature dendritic cells (DCs) during donor organ reperfusion in the recipient, which is driven by ischemia/reperfusion injury (IRI)-induced DAMPs. The interventional tools to accomplish this goal are outlined in greater detail: These involve prior administration of RCD inhibitors to the perfusate during NRP and/or NMP of the donor organ in order to prevent subsequent release of DAMPs from RCD types caused by IRI. Furthermore,—besides prevention of DAMP-promoted activation of intragraft donor- and recipient-derived iDCs during donor organ reperfusion—the potential role of already previously activated donor-derived DCs in triggering an alloimmune response could be inhibited by blocking their costimulatory molecules in advance during NRP or NMP. This scenario then would open a window of opportunity to induce allograft tolerance (26): In fact, when DCs are not activated by DAMPs, they fail to upregulate the necessary costimulatory signals required for T cell activation. And, notably, DCs presenting alloantigens in the absence of costimulatory signals were experimentally shown to result in the establishment of allograft tolerance (27–29). Indeed, the option of using RCD and DAMPs as therapeutic targets in NRP or NMP settings might hypothetically provide a chance for successful induction of allograft tolerance in recipients: The long-held dream of the transplant community to induce successfully allotolerance might become reality!

If targeted preclinical and clinical studies—along with the development of new drugs for clinical use—would provide proof-of-concept for the interventional strategies outlined in this review, this innovative therapeutic approach would need to be considered for application to all deceased donors, including DBD donors.

## 2 Introduction

In this Part 1, some background information is provided to understand why DAMPs and RCD should be utilized as biomarkers and therapeutic targets in NRP and/or NMP systems in organ transplantation. Thus, we first take a brief look at the world of DAMPs (covering their nature, classification, and function), followed by a concise description of mechanisms involved in the development of some forms of RCD. Following, a detailed presentation is given covering major periods of injury to which a donor organ is exposed to prior to the onset of innate alloimmune responses in the recipient after its transplantation. Throughout all these periods, various forms of RCD and their associated DAMPs accumulate in the donor organ and ultimately—after transplantation—elicit and orchestrate a robust innate alloimmune response in the recipient, resulting—without immunosuppression—in allograft rejection.

## 3 Decoding DAMPs and regulated cell death: key fundamentals at a glance

**Figure 2:**
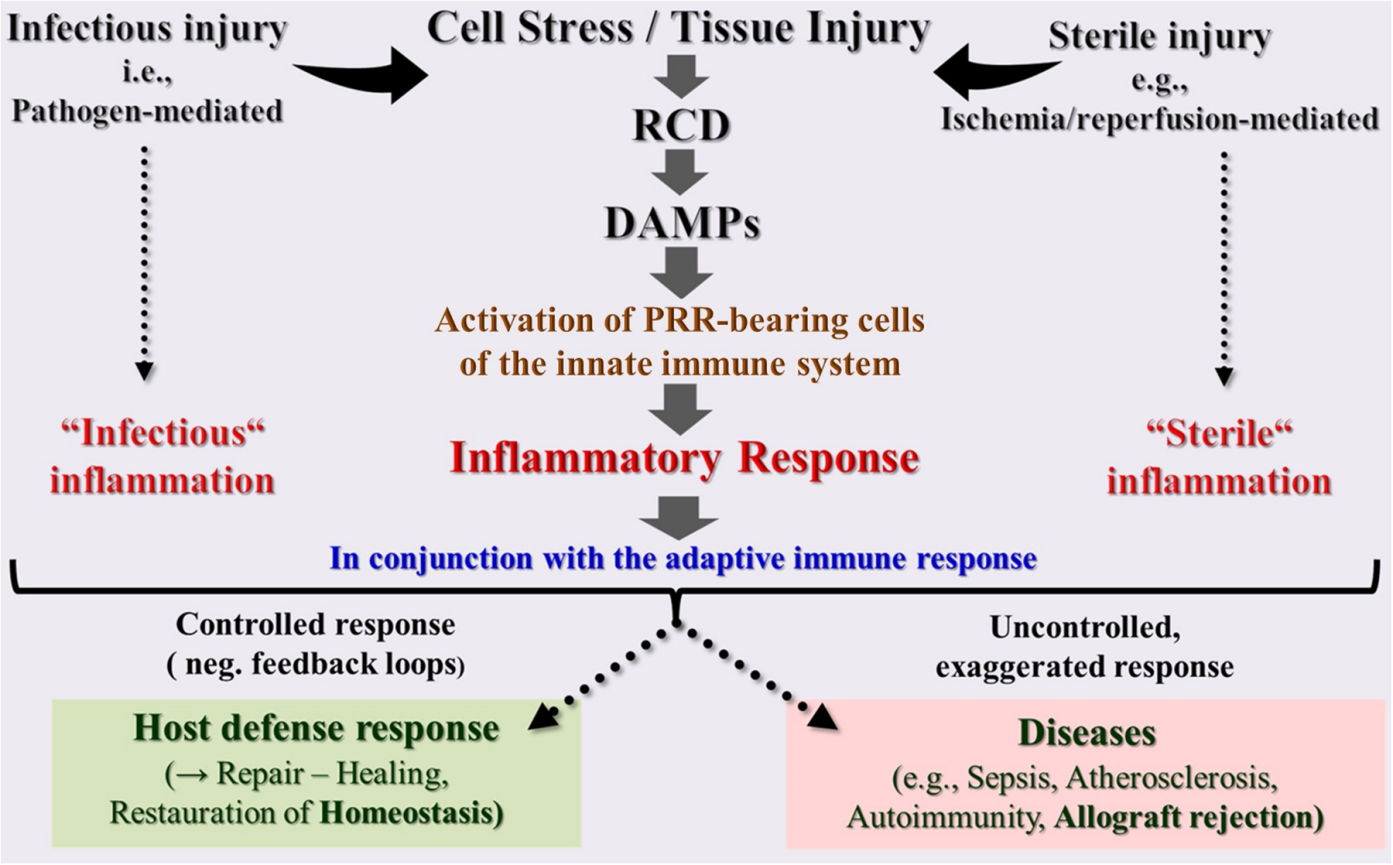
Schematic overview of the innate immune system as a highly sensitive organ of perception. This conserved first-line defense system, composed of somatic cells bearing pattern recognition receptors (PRRs), senses any cell stress or tissue injury and triggers either infectious or sterile inflammatory responses to maintain homeostasis. However, uncontrolled dysregulation of this system results in pathologies and diseases. DAMPs, damage-associated molecular patterns; PRRs, pattern recognition receptors; RCD, regulated cell death.

Undoubtedly, induction of RCD and the associated release of DAMPs are currently considered as unique fundamental Janus-faced processes. On the one hand, they are essential for restoring and maintaining cell and tissue homeostasis after both sterile and infectious injuries; on the other hand, if they occur in an uncontrolled and dysregulated manner, they can lead to a variety of life-threatening or even fatal human diseases (Figure 2). The issue is further complicated by the fact that both processes, when present and functioning in a controlled way, they are life-saving, while, when absent and not operating at all, they promote the growth of life-threatening tumors. From the wealth of studies published in an increasing amount and rapidity, some key aspects are briefly touched on in the following.

### 3.1 The world of DAMPs

#### 3.1.1 DAMPs in their role as friend and foe

DAMPs are molecules generated, exposed, and/or released in response to any cell stress and/or tissue injury, even from the slightest intra- and extracellular molecular disturbances. These unique molecules can act as both friend and foe—not only to humans but likely to all organisms living on our planet.

First and foremost, their evolutionarily determined function is to promote injury-induced defense responses to repair damaged tissue and maintain homeostasis. To accomplish these tasks, DAMPs trigger various innate immune responses that induce inflammation, cell proliferation, and fibrosis in a context-dependent manner. In fact, all organisms on our planet currently appear to use DAMPs to signal cell stress and tissue damage, regardless of whether they are sterile or infectious in nature (30).

However, there is also a dark side of DAMPs: Under uncontrolled and dysregulated conditions, DAMP-triggered, PRR-mediated responses can lead to pathologies and diseases, including the development of chronic inflammatory, autoimmune, and neurodegenerative disorders (31–33). But even worse: when DAMPs are emitted uncontrollably in excess and released locally and/or systemically in large quantities, such as in severe local or systemic tissue injury, an acute exaggerated local and/or systemic hyperinflammatory response may develop. This overshooting inflammatory response, when promoted by severe systemic injury, is observed, for instance, in septic COVID-19 patients in the intensive care unit (ICU) (34) or, when triggered by severe local injury, it presents, as outlined below, as a hyperinflammatory (innate) alloimmune response leading to acute allograft rejection (8) (Figure 2).

#### 3.1.2 Classification of DAMPs in a nutshell

**Figure 3:**
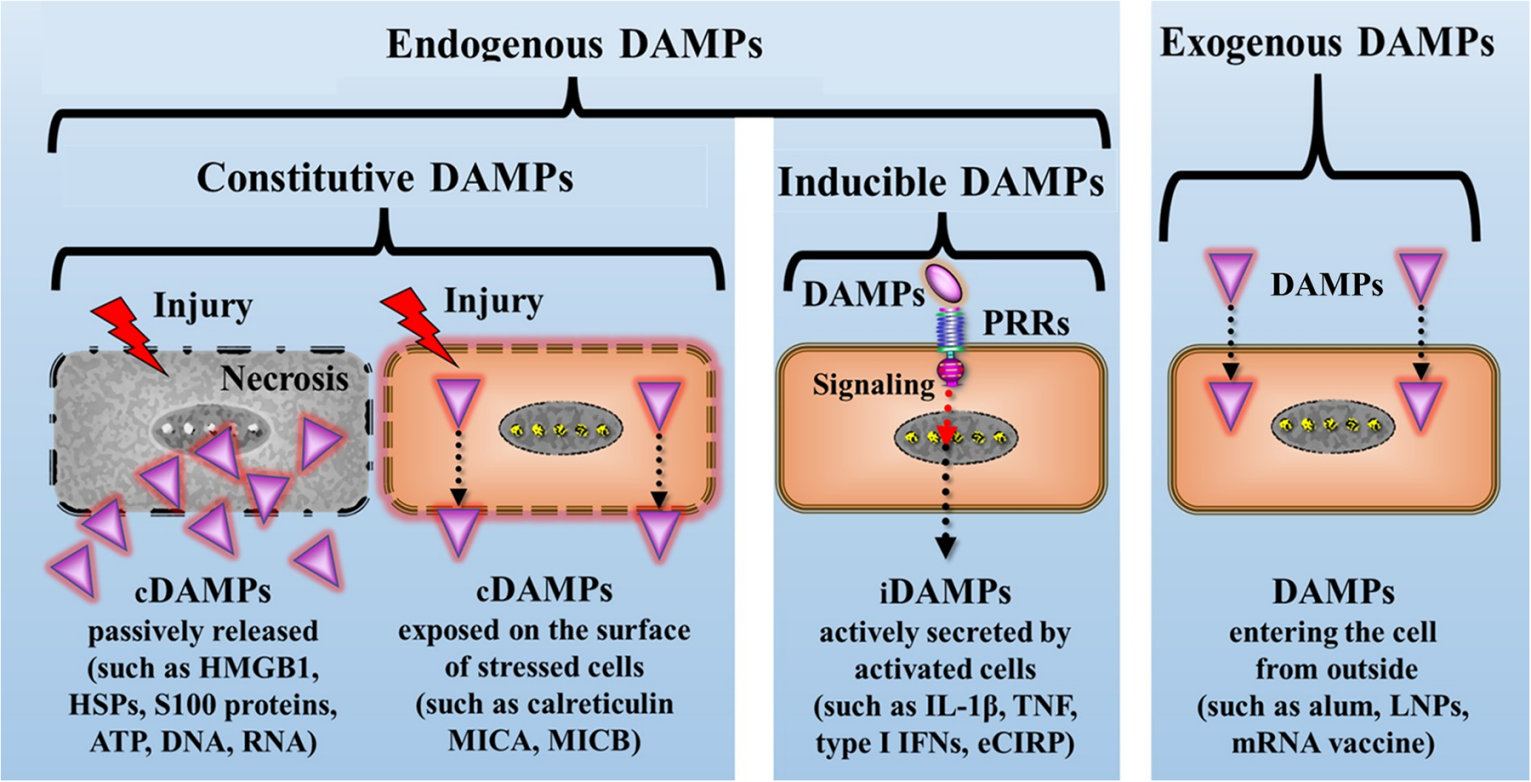
Simplified schematic diagram of the principal classification of DAMPs: A primary framework for categorizing DAMPs involves dividing them into four major groups. They are broadly classified as endogenous or exogenous DAMPs, with exogenous DAMPs representing molecules originating outside the host. Endogenous DAMPs are further subdivided into constitutive DAMPs (cDAMPs, either passively released or cell surface-exposed), and inducible DAMPs (iDAMPs) that are secreted by DAMP-activated innate immune cells. alum, aluminum hydroxide; eCIRP, extracellular cold-inducible RNA-binding protein; HMGB1, high mobility group protein B1; HSPs, heat shock proteins; IFNs, interferons; IL-1β, interleukin-1beta; LNPs, lipid nanoparticles; MICA, MICB, MHC class I chain-related protein A and B; mRNA, messenger RNA; PRRs, pattern recognition receptors; TNF, tumor necrosis factor.

Details on the classification of a wide spectrum of diverse DAMPs and an overview of the relevant literature can be found in (35) and, updated, in (36), as well as tables 1.1, 1.2, and 1.3 therein. According to this classification, the DAMPs can be roughly divided into 4 main categories (Figure 3**)**:

(I) Endogenous constitutively expressed native molecules (cDAMPs) such as high mobility group protein B1 (HMGB1), heat shock proteins (HSPs), S100 proteins, nucleic acids (NAs) such as mitochondrial DNA (mtDNA), nuclear DNA (nDNA), RNA, and histones as well as extracellular adenosine triphosphate (eATP), monosodium urate crystals, and cholesterol crystals. These molecules are generally released by necrotic cells, in particular, by cells succumbing various types of RCD (see below). Category I also encompasses cDAMPs that are exposed at the cell surface of stressed or dying cells including calreticulin (CALR) and major histocompatibility complex (MHC) class I chain -related molecules (MICs) such as MHC class I polypeptide-related sequence A and B (MICA and MICB), which have gained center stage as bona fide transplantation antigens (37).
(II) Endogenous constitutively expressed, but injury-modified molecules such as extracellular matrix compounds (ECMs) and cell-extrinsic modified DAMPs such as oxidation-specific epitopes (OSEs) as well as perturbation-induced, cell-intrinsically modified molecular patterns, which reflect cellular dyshomeostasis [here termed “dyshomeostasis-associated molecular patterns” or dysDAMPs, also called “HAMPs” (38)]. For example, eATP, via activation of the plasma membrane channel *purinergic P2X7 receptor* (P2X7R), leads to an efflux of K^+^ resulting in a decrease of intracellular K^+^ that generates such “molecular perturbation-reflecting” dysDAMPs.
(III) Endogenous inducible DAMPs (iDAMPs). These molecules are “newly made” by (DAMP)-activated cells upon cellular stress or tissue injury or even by cells undergoing (DAMP-promoted) RCD. They include native molecules operating as iDAMPs such as IIIA-2 DAMPs that are secreted by cDAMP-activated innate immune cells including interleukin-1 (IL-1) family members, tumor necrosis factor (TNF), type I interferons (IFN-I), *and extracellular cold-inducible RNA-binding protein* (eCIRP) that is secreted by stress-activated macrophages. Additionally, this category comprises modified molecules operating as iDAMPs such as anaphylatoxins (= complement fragments 3a and 5a (C3a and C5a). Importantly, this category also includes native molecules operating as counteracting **s**uppressing/inhibiting DAMPs [i.e., SAMPs, also denoted as “RAMPs” (39)] such as specialized pro-resolving mediators (SPMs) (40).
(IV) Exogenous DAMPs, which include molecules such as air pollution particles, aluminum salt, lipid nanoparticles, and *in vitro* modified mRNA vaccines.

As said, this is a rough classification of DAMPs which provides a valuable snapshot of their current descriptions, though it remains an evolving framework. Moreover, other mechanisms of DAMPs emission have been separately classified, such as their active secretion from stressed cells via extracellular vesicles. Such DAMPs include, but are not limited to, HMGB1, histones, HSPs, eATP, and NAs, among others [reviewed in (41)].

#### 3.1.3 DAMP-sensing receptors in triggering inflammation and immunity

It is now widely established that DAMPs trigger innate immune pathways by engaging PRRs on/in cells of the innate immune system, thereby promoting and amplifying subsequent efferent innate immune responses such as inflammatory reactions and—by engaging PRR-bearing antigen-presenting cells (APCs) such as DCs—instigating and shaping adaptive immune responses (42).

Pattern recognition receptors are formed on/in mobile sentinel and sessile innate immune cells, and it is conceivable that any viable cell, which is destined to defend against stress or damage, uses PRRs to sense and respond to DAMPs. The families of classical PRRs include Toll-like receptors (TLRs); NOD-like receptors (NLRs), whereby some members such as the *Nod-like receptor family pyrin domain containing 3* (NLRP3) form and activate inflammasomes; C-type lectin receptors (CLRs); and NA sensors, including endosomal sensors (NA-sensing TLRs), cytosolic DNA sensors (e.g., cGAS, AIM2), DNA-dependent activator of IFN (DAI) [also known as ZBP-1]), and cytosolic RNA sensors (e.g., RIG-I, MDA5, and LGP2) (42, 43). Moreover, DAMPs are reportedly sensed by nonclassical PRRs such as G-protein coupled receptors and ion channels (42), as well as by soluble humoral PRRs such as pentraxins (44).

**Figure 4:**
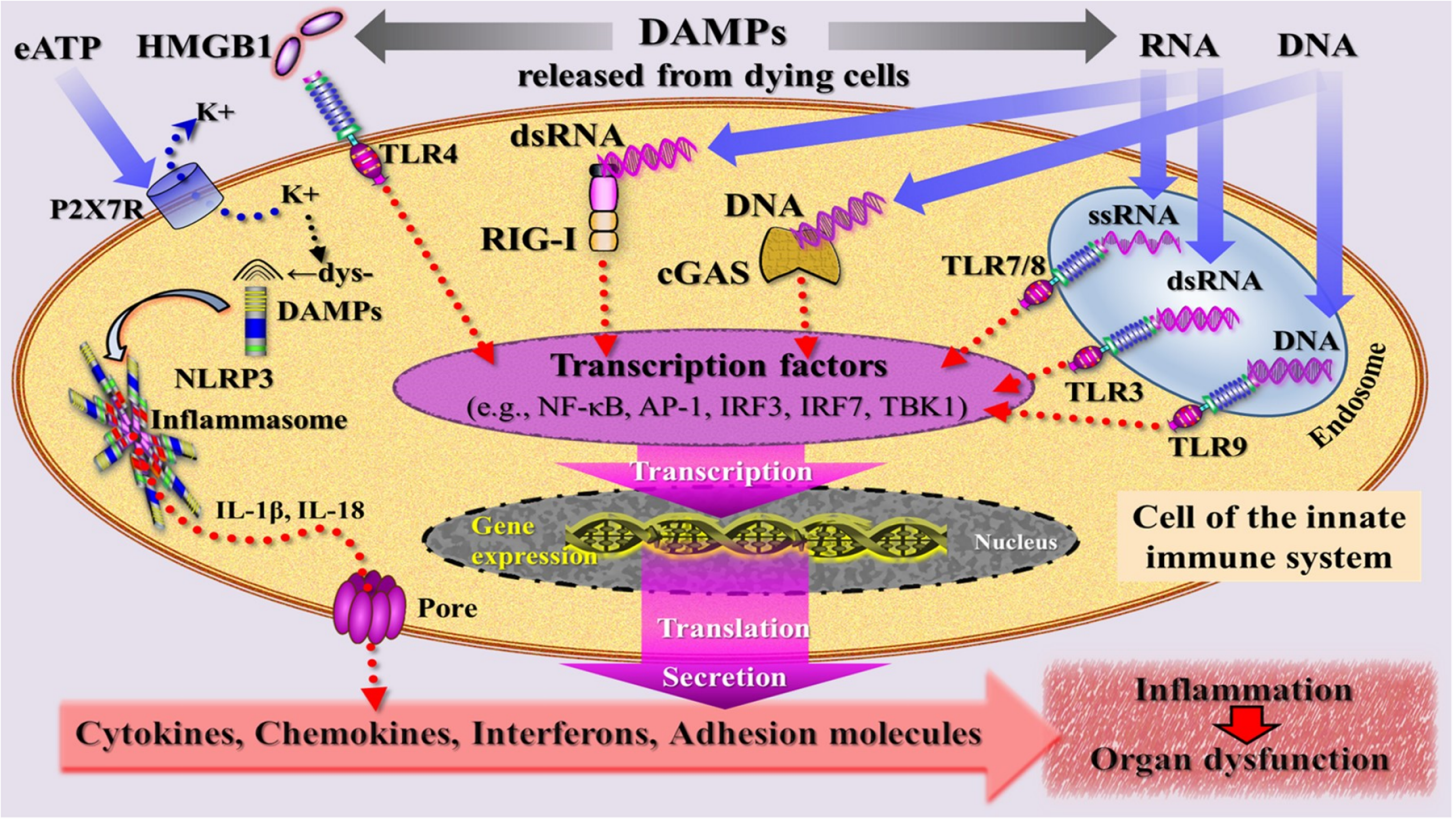
Simplified schematic diagram of a narrative model depicting injury induced, DAMP-triggered, pattern recognition receptor (PRR)-mediated trajectories that promote an inflammatory response associated with organ dysfunction. DAMPs [exemplified by high mobility group protein B1 (HMGB1), RNA, DNA] released by cells dying from regulated cell death are sensed by PRRs that are located at the plasma and endosomal membrane (TLRs) and in the cytosol (RIG-I, cGAS) of an innate immune cell. PRR-mediated signaling pathways (adaptor molecules not shown) lead—via transcriptional and translational processes (details not specified)—to secretion of inflammatory mediators that drive inflammation. The DAMP extracellular ATP (eATP)—via activation of the purinergic P2X7 receptor (P2X7R), induces dyshomeostatic DAMPs (dysDAMPs) that are sensed by NLRP3 receptor to contribute to the assembly of the inflammasome, which is associated with production of IL-1β and IL-18. These cytokines are released via transmembrane pores. AP-1, activating protein-1; cGAS, cyclic GMP-AMP synthase; dsRNA, double-stranded RNA; IL, interleukin; IRF3/7, interferon regulatory factor 3/7; NLRP3, nucleotide oligomerization domain (NOD)-like receptor protein 3; RIG-I, retinoic acid inducible gene I; ssRNA, single-stranded RNA; TBK1, TANK-binding kinase 1; TLRs, Toll-like receptors.

Engagement of DAMPs with these various recognition molecules on/in innate immune cells triggers signaling pathways converging on the production of proinflammatory cytokines and type I IFNs to mount robust efferent innate immune (= inflammatory) and fibrogenic/fibrotic defense responses (reviewed in (45–50) (Figure 4). Engagement of counteracting SAMPs with corresponding receptors drive inflammation-resolving responses (51), ensuring a well-regulated and controlled inflammatory process.

**Figure 5:**
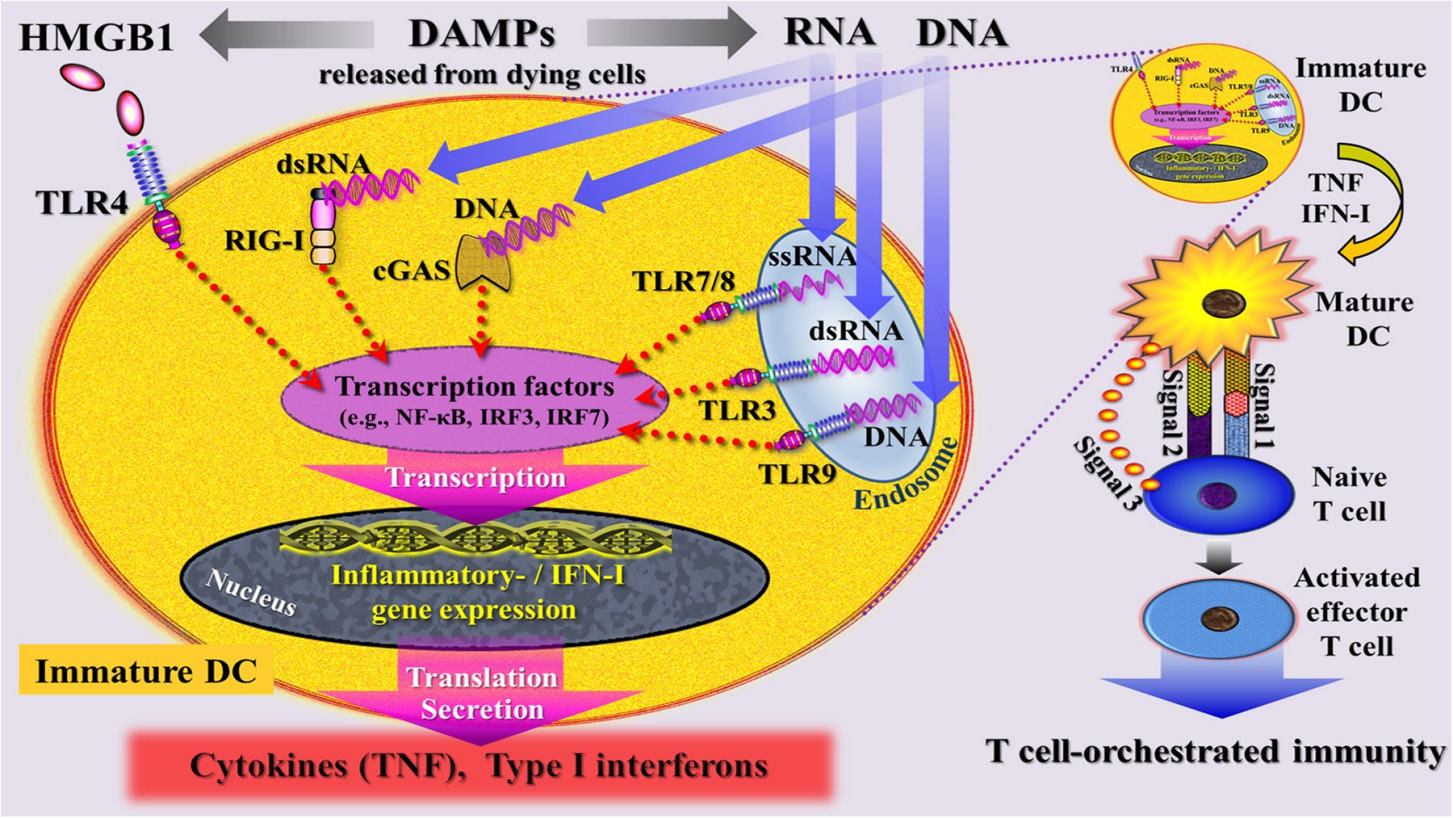
Simplified schematic diagram of a narrative model depicting injury induced, DAMP-triggered, pattern recognition receptor (PRR)-mediated trajectories that promote T cell-orchestrated immunity through activation of immature dendritic cells (DC) into mature DCs. DAMPs [exemplified by high mobility group protein B1 (HMGB1), RNA, DNA] released by cells dying from regulated cell death are sensed by PRRs that are located at the plasma and endosomal membrane (TLRs) and in the cytosol (RIG-I, cGAS) of an immature DC. These DAMP- triggered signaling pathways are believed to intersect at the level of interferon regulatory factors (IRFs) and nuclear factor kappa-light-chain-enhancer of activated B cells (NF- *κ*B), leading to transcriptional changes that drive—via gene expression—the maturation of DCs and promote the subsequent secretion of cytokines and type I interferons. signal 1: upregulation of peptide/MHC molecules; signal 2: upregulation of costimulatory molecules; signal 3: secretion of T cell-polarizing cytokines. cGAS, cyclic GMP-AMP synthase; DC, dendritic cell; dsRNA, double-stranded RNA; HMGB1, high mobility group protein B1; IFN-I, type I interferons; IRF7, interferon regulatory factor 7; RIG-I, retinoic acid inducible gene I; ssRNA, single-stranded RNA; TLRs, Toll-like receptors; TNF, tumor necrosis factor.

In the presence of altered self or nonself antigens such as alloantigens, tumor-associated antigens, or microbial antigens, injury-induced cDAMPs such as HMGB1, DNA, and RNA were shown to activate PRR-bearing iDCs into mature immunostimulatory DCs, thereby indirectly initiating and shaping adaptive immune responses (52–61). Specifically, cDAMPs released from dying cells, along with actively secreted iDAMPs, are considered critical factors in triggering the process of DC maturation and promoting subsequent production of proinflammatory cytokines. This process is discussed to involve the transcriptional regulation of genes encoding proinflammatory cytokines such as TNF and IFN-I (59, 60, 62, 63) (Figure 5).

### 3.2 Regulated cell death and some of its types

Regulated cell death or programmed cell death—a form of a cell demise triggered by external or internal stimuli—refers to the autonomous and well-ordered death of cells to control organismal development and repair/maintain internal homeostatic tissue stability upon stressful and/or injurious momenta.

Over the last decades, various types (also called forms) of RCD have been discovered, spanning from immunologically silent (non-lytic) apoptosis to highly proinflammatory/usually immunogenic forms of (lytic) regulated necrosis (RN) such as secondary necrosis following apoptosis, ferroptosis, necroptosis, and pyroptosis. In fact, RCD in the form of RN (RCD in this sense used throughout this article) is a hot topic in biomedical research on mechanisms involved in necroinflammation and adaptive immunity, as it serves as a major source for the release of both cDAMPs and iDAMPs [for reviews, see (8, 64–69)].

#### 3.2.1 The phenomenon of plasma membrane rupture

**Figure 6:**
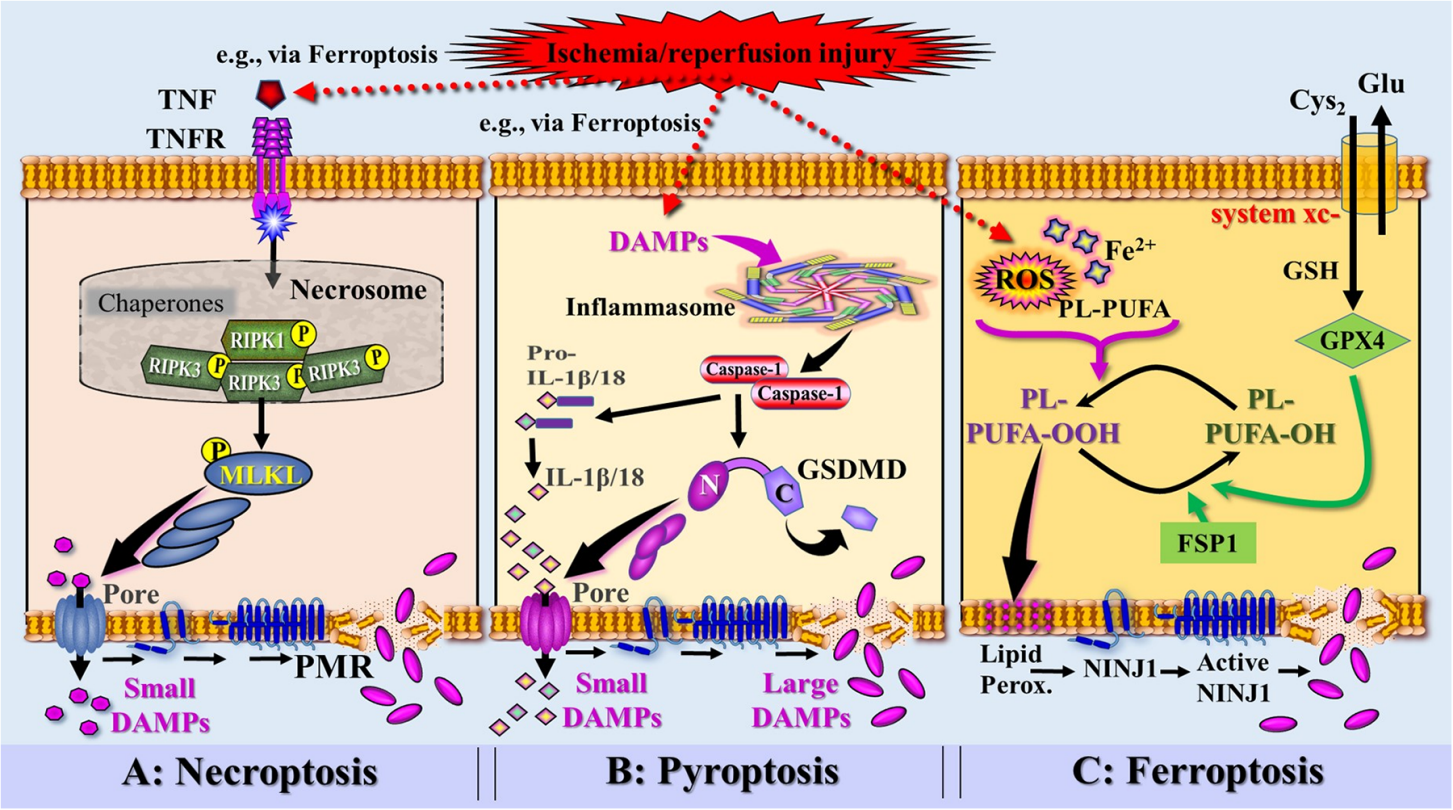
Simplified and rough schematic diagram illustrating a model of DAMPs released from injury-induced RCD types and controlled by ninurin-1 (NINJ1)-dependent plasma membrane rupture (PRM) (injury exemplified by ischemia/reperfusion injury). **(A)** Death receptor signaling [here exemplified by tumor necrosis factor receptor (TNFR) bound to TNF secreted by innate immune cells activated by DAMPs, e.g., released from ferroptosis] leads to the formation of the necrosome, which activates the receptor-interacting protein kinase 3 (RIPK3). RIPK3 phosphorylates the molecule MLKL, which forms pores to instigate a PMR-associated necroptotic cell death, which is (partially) dependent on NINJ1. **(B)** Perception of DAMPs (e.g., released from ferroptosis) triggers the canonical inflammasome pathway and the activation of the inflammatory caspase-1. Caspase-1 is capable to cleave pro-IL-1β and pro-IL-18 into the mature cytokines. Activation and assembly of the inflammasome results in activation of Gasdermin D (GSDMD), whereby the caspase-1-cleaved N-terminal of GSDMD oligomerizes in membranes to proceed to final pores that release small DAMPs including the iDAMPs IL-1β and IL-18. GSDMD pores also drive the pyroptotic cell death associated with PMR that requires NINJ1 activation. **(C)** Accumulation of ischemia/reperfusion injury-induced reactive oxygen species (ROS) and iron (Fe2^+^) generate phospholipid hydroperoxides (PL-PUFA- OOH) leading to induction of ferroptosis. Ferroptosis suppressor protein 1 (FSP1) and glutathionperoxidase 4 (GPX4)—requiring the cofactor glutathione (GSH)—counterbalance the ferroptotic pathway by reducing PL-PUFA-OOH into lipid alcohols (PL-PUFA-OH). Phospholipid peroxides cause plasma membrane lipid peroxidation associated with permeabilization and the formation of NINJ1, which progresses into large NINJ1 oligomers that execute PMR. **Source:** Ramos et al. Ref. 69; C, C-terminal domain of GSDMD; Cyto-c, cytochrome c; Glu, glutamine; Lipid Perox., lipid peroxidation; MLKL, mixed lineage kinase domain like pseudokinase; N, N-terminal domain of GSDMD.

As research on RCD has progressed and novel mechanisms orchestrating multiple cell death pathways have been discovered, a molecularly oriented classification of various cell death types was proposed, focusing on the mechanistic and essential aspects of the process (65). Despite the differences in the signaling mechanisms of each cell death pathway observed in types of RN, the underlying lytic cell death typically culminates in a subsequent plasma membrane rupture (PMR) (68). For many years, PRM was regarded as a passive process attributable to osmotic pressure buildup, followed by cell swelling. This view has recently changed with the discovery of Kayagaki et al. (70) demonstrating that the plasma membrane resident protein *ninjurin-1* (NINJ1) is essential for PMR, indicating that membrane rupture is not simply a passive event but a tightly regulated, active process.There is growing evidence confirming that NINJ1 is critical for PMR (although not the only mediator) during a range of RCD modalities, including necroptosis, pyroptosis, ferroptosis, cuproptosis, cell death induced by pore forming toxins, and even secondary necrosis of apoptotic cells [reviewed in (71)] (Figure 6).

Among the best-studied modalities are ferroptosis, necroptosis and pyroptosis, all of which qualify as “immunogenic cell death (ICD)” because they release DAMPs as a result of PMR. They are addressed below in a simplified and concise manner. Other forms of regulated necrosis, such as autophagy, apoptosis→secondary necrosis, NETosis, parthanatos, entosis, cuproptosis, and panoptosis have been described (65, 67) but are not discussed in detail here.

#### 3.2.2 Ferroptosis

Ferroptosis is classified as a type of RCD that is triggered by an iron-dependent rise in lipid peroxidation within cellular membranes. The mechanism of ferroptosis is complex and morphologically, genetically, and biochemically distinct from other forms of RCD. Thus, the dying process is regulated by the interaction of multiple metabolic pathways, including those governing iron metabolism, lipid peroxidation, and antioxidant systems (72–74). Interestingly, glucocorticoids and siRNA treatment have been demonstrated to sensitize to ferroptosis (75, 76). Ferroptosis is initiated by the depletion of intracellular glutathione that under homeostatic conditions, neutralizes free hydroxyl radicals and reactive oxygen species (ROS) and thus protects cells from oxidative stress and lipid peroxidation. Ferroptosis is additionally instigated by the depletion of antioxidative enzymes, including glutathione peroxidase 4 (GPX4) and ferroptosis suppressor protein 1 (FSP1), both reducing lipid peroxidation. This decreased activity of these cell's natural antioxidative defense capacities results in the accumulation of unmetabolized lipid peroxides and the production of high levels of ROS and hydroxyl radicals. The resulting lipid peroxidation process—as the key mechanism of ferroptosis—then leads to ion fluxes, organelle failure, damaged cell membranes, and cell swelling, which, in turn, lead to PMR and cell death (77).

Of note, NINJ1, as mentioned above, has also been proposed by Ramos et al. (78) to promote PMR during ferroptosis. In this article, the authors concluded that their data support a model for ferroptotic cell lysis in which lipid peroxidation results in increased plasma membrane tension causing the opening of mechanosensitive ion channels and an ion disbalance followed by activation of NINJ1 [for mechanosensitive ion channels perceiving DAMPs, see (79)]. Active oligomeric NINJ1 then induces a loss of membrane integrity and, at later time points, eventually complete membrane rupture and cell lysis (80, 81) (Figure 6). As a consequence of NINJ1-dependent PMR, ferroptotic cells begin to release intracellular contents, among them many DAMPs capable of driving inflammation and—in the context of this article- shaping adaptive immunity.

#### 3.2.3 Necroptosis

Necroptosis refers to a distinct form of RCD triggered by a variety of extracellular stimulating ligands that engage death receptors on the cell surface. The full details of our current understanding of necroptotic pathways can not be outlined here; instead, only some key points will be briefly touched upon [reviewed in (68, 73, 82, 83)].

Necroptosis can be initiated by *tumor necrosis factor receptor* (TNFR), PRRs, including TLR3 and TLR4, NLRs, and RLRs, INF-α receptors, and DAI (ZBP-1). At the beginning of the necroptosis-inducing trajectory, stress signaling ligand ↔ cognate death receptor interactions include FAS ligand (FASL) with FAS, TNF with TNF receptor 1 (TNFR1), and TNF-related apoptosis-inducing ligand (TRAIL) with TRAILR1 or TRAILR2. Beyond death receptors, the necroptosis machineries can also be engaged by TLR3, TLR4, or DAI (ZBP1) as well as IFN-α receptors.

Under normal conditions, those stress signals activate caspase-8 initiating apoptosis. However, when caspase-8 activity is suppressed, receptor-interacting protein kinase 1 (RIPK1) and RIPK3 are activated, forming a complex called the necrosome. The necrosome promotes the oligomerization and phosphorylation of *mixed lineage kinase domain-like protein* (MLKL), whose oligomeric form translocates from the cytosol to the plasma membrane, leading to the formation of membrane pores and instigating a lytic form of cell death that is partially also dependent on NINJ1 (69, 70, 84, 85). NINJ1-mediated pores are formed that increase plasma membrane permeability, thereby causing membrane rupture (Figure 6). Notably, while pore formation already permits the release of iDAMPs such as TNF, IFN-I, and IL-1β, the rupture of the plasma membrane enables the release of large cDAMPs such as HMGB1 as well.

#### 3.2.4 Pyroptosis

Pyroptosis is generally divided into two major trajectories, the canonical inflammasome pathway mediated by caspase-1 and the noncanonical inflammasome pathway mediated by caspase-4/5 and caspase-11. As a highly inflammatory form of RCD, pyroptosis is instigated by the activation of PRRs in response to DAMPs including the abovementioned dysDAMPs, leading to the assembly of inflammasomes [reviewed in (66, 67, 86)]. Inflammasomes such as the NLRP3 and the AIM2 inflammasome are cytoplasmic multiprotein complexes that consist of PRRs, adaptor proteins, and caspases (caspase-1, -4, and -5 in humans, and caspase-1 and -11 in mice) [reviewed in (87)]. These inflammatory enzymes cleave members of the gasdermin (GSDM) families to produce an N-terminal gasdermin fragment that forms membrane pores (note that humans have six gasdermin family members (GSDMA, GSDMB, GSDMC, GSDMD, GSDME, and GSDMF). In addition, the enzymes proteolytically process pro-IL-1β and pro-IL-18 into the mature signaling IL-1β and IL-18, which are released through these pores. Mechanistically, it is currently discussed that these pores in the plasma lead to osmotic swelling through water influx, ultimately culminating in NINJ1-dependent PMR, which enables the release of large DAMPs (68, 70) (Figure 6).

### 3.3 Conclusion

To sum up, this chapter provides merely a brief excerpt of the expanding research field exploring the spectrum of DAMPs and the phenomenon of cell death as a tightly regulated autonomous cellular response to severe, insurmountable stress. The topic can also be framed in the context of necroinflammation, whereby cell death pathways serve as its origin and various classes of DAMPs as its consequences. Necroinflammation can thus be defined as innate immune responses to necrosis within a living organism (64). Accordingly, whenever a cell undergoes RCD, its intracellular content is released as DAMPs, which engage with PRR-bearing cells of the innate immune system to trigger necroinflammation.

Notably, the scenario can also be interpreted as an inherent intertwining bio-entity of RCD and DAMPs (in the following often marked as RCD→DAMPs) serving as an evolutionarily conserved and highly effective mechanism for initiating host defense against any injury. In the context of organ transplantation, this bio-entity of RCD→DAMPs can then be seen as a potent mechanistic trigger that initiates an innate alloimmune response to any form of allograft injury—driving allograft rejection not primarily as a reaction to “foreignness” but as a targeted response to injury.

## 4 Critical momenta of injury: unraveling the forces shaping donor organ immunogenicity

According to the principle of the danger/injury model, it is currently understood that any severe insult to an organ promotes the development of RCD with subsequent release of DAMPs. Per definition, every donor organ is unavoidably exposed to a series of damages until it is implanted and reperfused in the recipient. But also after reperfusion and the onset of the innate alloimmune response in the recipient, injurious events such as acute and chronic allograft rejection and infections may lead to RCD→DAMPs (12) (see Figure 1).

However, here, only distinct periods of injury to which a donor organ is exposed to before transplantation into the recipient are of interest. Thus, initial damage to the organ, along with the induction of RCD and the release of DAMPs, may have already occurred during an earlier traumatic or cardiovascular accident. This is followed by further release of DAMPs from cells undergoing RCD under conditions of DBD or DCD. Subsequent damaging events may continue during organ preservation procedures. Ultimately, the accumulation of DAMPs during these periods of injury reflects the growing immunogenicity of the organ. A few aspects of these scenarios are sketched in the following.

### 4.1 Potential traumatic events of the donor before admission to the intensive care unit

For DBD donors, first severe damage to a potential donor organ may already occur during a previous traumatic accident, especially if associated with hypoxia and shock. Accordingly, a significant emission of DAMPs has been reported in polytrauma or solid organ trauma, potentially released from various forms of RCD, though this has not been systemically investigated [with a few exceptions in the context of necroptosis (88, 89) and pyroptosis (90)]. For example, in polytrauma patients, a large spectrum of cDAMPs, including HMGB1, eATP, histones, S100A proteins, CIRP, cell-free DNA and RNA as well as iDAMPs such IL-1 and IL-33 has been demonstrated (36, 91). Similarly, in severe burns and shock including hemorrhagic shock, some of those DAMPs have been described (92–94). By contrast, more insights are known about the occurrence of RCD and emission of DAMPs during DBD conditions, which will be alluded to in more detail below.

Notably, DCD donors may also have suffered a previous traumatic accident. However, as a separate category of donors, other harmful episodic events may have occurred due to severe comorbidities, particularly cardiovascular diseases, which often go along with hypoxic episodes. Overall, it appears to be challenging to accurately evaluate the nature and severity of these prior events in retrospect, particularly in relation to the identification of RCD and DAMPs.

### 4.2 Donation after brain death donor conditions

The induction of RCD as well as the release of DAMPs have not been systematically studied in organ donors under brain death (BD) conditions. However, acute cerebral pathologies such as traumatic brain injury (TBI) and ischemic or hemorrhagic stroke that may ultimately result in catastrophic BD are increasingly being used as research subjects to explore the role of RCD and DAMPs in promoting acute cerebral inflammation. The pathophysiology of brain injury associated with both pathologies is complex and is briefly outlined here.

#### 4.2.1 Primary and secondary brain damage

Typically, in TBI and hemorrhagig stroke, it is essential to distinguish between primary and secondary brain injury [reviewed in (95–97)]. Primary brain injury represents local/focal damage caused, for example, by epidural and subdural hematoma, brain contusion, and focal intracranial hemorrhage. This injury is caused by direct damage to neural tissue via external mechanical forces and determines mainly the patient's outcome. Incidentally, DAMPs are also generated and emitted. Of note, the intense DAMP-orchestrated neuroinflammation occurring during this acute phase triggers a disruption of the blood-brain barrier (BBB) (98, 99) and promotes additional neuronal injury.

Subsequently, a secondary brain injury developes that is the result of a highly complex pathogenesis that involves numerous processes. These include (primary) DAMP-promoted innate immune pro-oxidative/pro-nitrous and proinflammatory processes, which are associated with increasing cerebral edema and ischemia and ultimately converge to and culminate in cell death pathways. Indeed, all these injury-associated pathophysiological processes are known to contribute to the death of cerebral cells, such as astrocytes, oligodendrocytes, and microglia, as evidenced by the manifestation of various forms of RCD (which are considered hallmarks of secondary brain injury) [also see (100, 101)].

The pathophysiology of ischemic stroke is somewhat different and more complex [for reviews, see (100, 102, 103)]. Notably, during the acute phase, the primary ischemic injury drives an intense neuroinflammatory response that is also associated with a breakdown of the BBB and secondary damaging processes (98, 104).

In line with this pathological context—and on “borrowing” findings from studies on TBI and stroke-, the following depictions of the role of DAMPs and RCD under BD conditions should be interpreted accordingly.

#### 4.2.2 Development of regulated cell death types

As already touched above, compelling evidence indicates that types of RCD are involved in the various phases of TBI and stroke, including ferroptosis, necroptosis, and necroptosis.

##### 4.2.2.1 Ferroptosis

A key role in these scenarios can be attributed to ferroptosis: For example, emerging evidence has been reported indicating a substantial role of ferroptosis in TBI (105). In addition, compelling evidence was provided implicating ferroptosis as a mechanism driving neuroinflammatory processes in ischemic and hemorrhagic stroke *in vivo* (106). Investigating the mechanisms underlying neuronal death in ischemic stroke, considerable evidence has been gathered indicating that ferroptosis is triggered by events of excitotoxicity, oxidative stress, and inflammatory responses (107).

##### 4.2.2.2 Necroptosis

Necroptosis is also a key feature in the pathological processes of TBI and stroke. Thus, in a review of existing information on the mechanism by which necroptosis participates in TBI, ligand-receptor-induced necroptosis was shown to be executed by the cell death pathway composed of RIPK1, RIPK3, and MLKL (108, 109). In addition, there is some evidence for the participation of necroptosis in ischemic stroke. As reviewed (107, 110), TNF operates as a trigger of this RCD type, while several proteins are identified as necroptosis regulators by modulating the activities of RIPK1, RIPK3, or MLKL.

##### 4.2.2.3 Pyroptosis

Increasing evidence from studies using mouse models suggests that TBI—probably in the secondary phase of injury- induces pyroptosis, with caspase-1 and GSDMD playing a significant role in neuroinflammation (111–113). In ischemic stroke, various studies on mouse and rat models have demonstrated the presence of pyroptosis-specific markers in the brain tissue after stroke, such as NLRP3, caspase-1/11, and GSDMD (114). Remarkably, in clinical studies, NLRP3 inflammasome components and IL-1β and IL-18 were found to be upregulated in postmortem brain tissue samples from patients with stroke (115). Moreover, pyroptosis has also been identified as a critical contributor to neuroinflammation in hemorrhagic stroke (114, 116).

In sum, these first observations on the role of forms of RCD in TBI and stroke are in support of the concept that this form of cell death serves as a critical source for DAMPs, which trigger necro-neuroinflammatory responses involved in the pathogenesis of these life-threatening diseases.In this sense, the integrated function of RCD-derived DAMPs may be considered as the initial stimulus that ultimately can lead to BD.

#### 4.2.3 Generation and emission of DAMPs in brain injury

Anecdotally, the first DAMP (though not yet termed DAMP at that time) detected in biopsies of cold-stored kidneys removed from DBD donors was identified as HSP70 (117). Subsequently, further reports on the detection of cDAMPs and iDAMPs in organs removed from DBD donors were published, for example, HMGB1 (118) and mtDNA (119).

Of note, in cases of TBI-induced as well as stroke-associated brain injury that ultimately lead to BD, DAMPs can be distinguished at two distinct locations: (1) DAMPs generated *intracerebrally* in the course of the primary injurious processes, and (2) DAMPs primarily generated *in the periphery* under BD-mediated pathological conditions such as hypoxia, metabolic disturbances, and other injurious processes.

##### 4.2.3.1 Intracerebral generation and emission of DAMPs

When regarding the intracerebral emission of cDAMPs and iDAMPs as observed in TBI and stroke, two waves of their generation can be conceptually discussed: one in the course of primary (mechanical/ischemic) brain injury released from cerebral cells succumbing from ACD or RCD due to initial, TBI- or stroke-mediated local/regional insults, and another during secondary brain injury released from neuronal cells undergoing RCD [for details, see (96, 100)]. In the worst case, e.g., in severe TBI or stroke, these DAMPs may in turn further promote cell death pathways resulting in types of RCD, thereby creating a vicious circle of DAMPs emission that ultimately leads to irreversible BD.

Regardless of the origin of DAMPs during primary or secondary brain injury, cDAMPs (such as HMGB1, HSPs, S100 proteins, eATP, mtDNA, and RNA), as well as iDAMPs (such as TNF and members of the IL-1 family), have been detected in studies on experimental TBI models and, less frequently, in patients with TBI. Similar findings have been reported from studies on stroke models and stroke patients, in which cDAMPs (including HMGB1, HSPs, S100 proteins, eATP, and histones) and iDAMPs (such as TNF and members of the IL-1 family) have been demonstrated [for further reading, see (96, 120)].

Of note, these DAMPs not only drive catastrophic neuroinflammatory pathways in the brain but can also cross the compromised, disrupted BBB to travel into the periphery to reach and affect—within minutes after injury—remote organs, that is, potential transplants! For this property, these DAMPs have been designated as circulating DAMPs. For example, poststroke circulating DAMPs were found to comprise a diverse group of molecules including cDAMPs such as HMGB1, S100A8/A9 proteins, HSPs, and iDAMPs such as TNF, IL-1β, and peroxiredoxins (121). Similar mechanisms of the role of DAMPs in propagating neuroinflammation to the systemic compartment have also been reviewed for TBI (122).

##### 4.2.3.2 Peripheral generation and emission of DAMPs under conditions of brain injury

Apart from the scenario of intracerebrally emitted DAMPs to drive—as “circulating DAMPs”—peripheral systemic inflammation, the primary emission of DAMPs in peripheral remote organs is also discussed to contribute to this phenomenon. In fact, for more than 20 years, it has been known that BD in organ donors induces substantial circulatory, hormonal, and metabolic changes associated with a systemic inflammatory response (123). Emerging evidence now indicates that these BD-mediated pathophysiological events such as hypoxia, oxidative stress, oxidative stress-induced stress of the endoplasmic reticulum (ER), and disturbed metabolism—probably via emission of DAMPs—activate the peripheral innate immune system, which contributes to the development of an acute systemic autoinflammatory syndrome (124–127).

#### 4.2.4 Activation of the donor's innate immune system in DBD

##### 4.2.4.1 Brain death-associated systemic (auto)inflammatory response syndrome

The abovementioned scenario of brain injury-elicited disruption of the BBB is the key momentum for the long-known understanding that alerting the periphery is a typical response of the CNS to injury. Indeed, the neuroinflammatory response following TBI or stroke is not confined to the CNS but extends beyond it, affecting remote organs outside the brain (98, 120). As recently reported, it is the DAMPs that have been identified to trigger these peripheral systemic innate immune responses following stroke and TBI (121, 122)**.** Of note, however, a key challenge for future research lies in determining the specific roles and relative contributions of circulating DAMPs originating from the brain vs. those locally emitted during peripheral pathophysiological events in activating the organismal innate immune system.

Regardless of the origin of DAMPs, their ability to induce and orchestrate a clinically manifest robust systemic inflammatory response syndrome (SIRS) is evidenced by the demonstration of upregulated PRRs triggering signaling molecules, inflammatory mediators such as cytokines, chemokines, upregulated chemokine receptors, complement fragments, and adhesion molecules (128, 129) [for review, see (8)].

But again: a significant challenge for future research is to determine the relative contributions of inflammatory mediators originating from the brain vs. those produced locally during peripheral pathophysiological events in the development of a SIRS. Notwithstanding, this inflammatory complication has also been observed to affect organs from DBD donors (123). However, it is important to mention that the results of a recent study on a cohort of donors with brain death revealed no evidence of a progressive proinflammatory cytokine storm (130).

##### 4.2.4.2 Brain death-associated activation of donor dendritic cells

In the context of the activation of the donor's innate immune system, analyses of DC subsets in the human spleen obtained from brain-dead organ donors are of interest. Thus, in a first study on human spleen fragments obtained from such deceased donors, the results showed the presence of both conventional/myloid DCs and plasmacytoid DCs at different stages of maturation (131). In similar subsequent investigations, it was found that activation markers (e.g., CD80) on spleen DCs were elevated, although their expression levels were lower than on fully activated DCs. In addition, it was observed that spleen DCs from brain-dead donors were able to weakly elicit T cell proliferation (132).

Certainly, further targeted experiments are needed to gain a more precise understanding of the activation status of DCs in DBD donors.

#### 4.2.5 Résumé: the immunogenicity of organs from DBD donors

**Figure 7:**
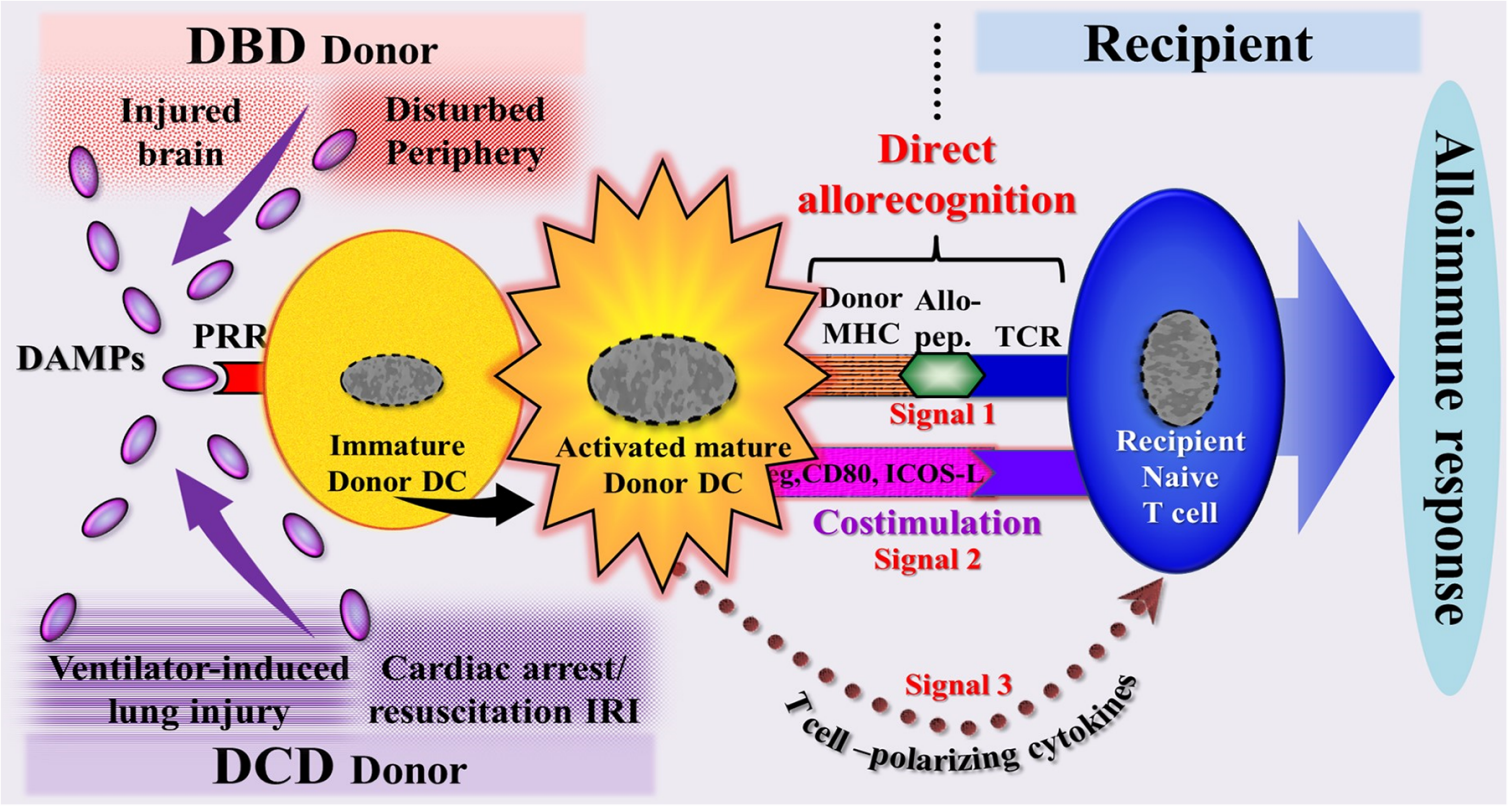
Schematic diagram of a model illustrating the DAMP-triggered, donor dendritic cell (DC)-mediated direct allorecognition process resulting in an alloimmune response of the recipient. This scenario reflects the immunogenicity of a potential allograft, as demonstrated by a powerful immune response of the recipient to the deceased donor's alloantigens. In organs from DBD donors, DAMPs originate (1) from injury-induced RCD occurring during brain death conditions in the cerebrum and migrating to the periphery (intracerebrally generated → circulating DAMPs) and (2) from the periphery (DAMPs emitted primarily in the periphery during pathophysiological events). In organs from DCD donors, DAMPs are supposed to originate mainly from injury-induced RCD occurring during lung ventilation and cardiac arrest→successful resuscitation -induced ischemia/reperfusion injury. Allo-pep., allogeneic peptide; DBD, donation after brain death; DCD, donation after circulatory death; ICOS-L, inducible costimulator-ligand; IRI, ischemia reperfusion injury; MHC, major histocompatibility complex; PRR, pattern recognition receptor; TCR, T cell receptor.

Organs from DBD donors destined for transplantation contain DAMPs, exhibit inflammation, and are therefore considered immunogenic organs. As discussed, these DAMPs originate form both cerebral and peripheral sources. It is essential, however, to note that their generation and release from necrotic cells are contingent upon an intact organismal metabolism and sustained blood flow, conditions characteristic of ventilated DBD donors. Indeed, these donor organs are packed with cDAMPs and iDAMPs and likely DAMP-activated donor-derived DCs, which are co-transplanted into the recipient, thereby conferring immunogenicity (Figure 7). Notably, as will be described in Part 2, donor-derived DCs play a crucial role in mediating the phenomenon of direct allorecognition—a key momentum driving the development of alloimmune-mediated acute rejection within the first three months posttransplant.

### 4.3 Donation after circulatory death conditions

The shortage of organs remains the primary obstacle to the expansion of transplant therapies globally. In recent decades, there has been a resurgence of interest in donations from individuals whose death is determined by circulatory and respiratory criteria, known as DCD. Consequently, the use of organs from DCD donors has steadily increased in the USA (133) and, to a lesser extent, in some European countries (134). Potential DCD organ donors are currently categorized into groups known as the Maastricht categories, which were updated in 2013 (135). According to these modified criteria, they can be broadly classified into uncontrolled DCD (uDCD) and controlled DCD (cDCD), but in total into 6 categories, with some subgroups being distinguished (135) (Table 1). The injuries to organs from DCD donors differ from those of DBD donors and warrant a brief description here.

**Table 1:** The modified Maastricht classification of DCD [Thuong et al. (135)].

Categories	Subcategories	Definition of end-of-life situation
Category I: Uncontrolled (uDCD)	Found dead IA. Out-of-hospital	Referring to irreversible circulatory death out of hospital without any attempt of resuscitation by a life-medical team
	Found dead IB. In-hospital	Referring to irreversible circulatory death in-hospital without any attempt of resuscitation by a life-medical team
Category II: Uncontrolled (uDCD)	Witnessed cardiac arrest IIA. Out-of- hospital	Referring to irreversible circulatory death with witnessed cardiac arrest out of hospital despite attempted resuscitation (= unsuccessful resuscitation)
	Witnessed cardiac arrest IIB. In-hospital	Referring to irreversible circulatory death with witnessed cardiac arrest in-hospital despite attempted resuscitation (= unsuccessful resuscitation)
Category III: Controlled (cDCD)	Withdrawal of life-sustaining therapy	Referring to (expected) irreversible circulatory death following planned withdrawal of life-sustaining-therapy in the hospital
Category IV: Uncontrolled/Controlled (uDCD/cDCD)	Cardiac arrest while life → brain dead	Referring to irreversible circulatory death with cardiac arrest during or after criteria for brain death completed in the hospital

#### 4.3.1 Ischemia times in DCD and their impact on Inferior transplant outcomes

Compared to DBD, the main disadvantage of DCD (whether controlled or uncontrolled) is the prolonged primary warm ischemia times, which are associated with (time-dependent) anoxic/hypoxic damage to the donor organ. To make matters more challenging, warm ischemia time is often unknown and can only be approximately estimated in uDCD, whereas it can be precisely calculated in cDCD (135, 136). For example, in cDCD, warm ischemia time after withdrawal of life-sustaining therapies has been defined as a period of functional warm ischemia time (fWIT) starting when the systolic blood pressure drops down below 50 mmHg following treatment withdrawal, proceeding to circulatory arrest (asystole), followed by the declaration of death after adhering to a “no-touch” period (2 min-20 min, usually 5 min), and ending with cooling perfusion after transfer to the theatre and completed laparotomy/thoracotomy (135, 136).

Empirically, the utilization of organs from DCD donors has been shown to be linked to inferior posttransplant outcomes compared to those from DBD donors (14). In kidney transplantation, for example, there is a higher risk of delayed graft function (DGF), primary nonfunction (PNF), and an increased risk of graft loss in the first year posttransplant, although DCD kidneys reportedly provide similar long-term graft survival, function, and patient survival when compared to kidneys procured from DBD donors (137, 138). Similarly, in liver transplantation, an increased risk of early allograft non- or dysfunction and the development of biliary complications (ischemic cholangiopathy) has been reported (139, 140).

It soon became evident that the underlying cause of the poorer results was the extended warm ischemia time, an observation reported, for example, for DCD kidneys (141, 142) and DCD livers (143, 144). In the context of DCD, overall ischemia times are longer as fWIT in cDCD, yet variable in uDCD depending on technical procedure, success, and nature of resuscitation efforts. This ischemic period is characterized by ischemic injury, the accumulation of toxic metabolites, and depletion of metabolic substrates, resulting in end-organ dysfunction (for details, see below, Section 5.1.2**)**.

Further studies in DCD donors showed that the prolonged warm ischemic phase increases the susceptibility of DCD organs to damage occurring during subsequent cold storage. For example, cold-stored kidneys from DCD donors were shown to be associated with poorer graft survival than those from DBD donors (145). Moreover, the acceptance of prolonged warm ischemia times in DCD donors (as well as in extended criteria DBD donors) pave the way for an increased vulnerability of donor organs to subsequent—intensified—IRI in the recipient (146). As a reason for this, accumulation of metabolic products have been made responsible, whereby accumulation of succinate seems especially to contribute later to increased IRI after reperfusion of the donor organ in the recipient (147). And it is this scenario that has driven the development of dynamic preservation techniques, such as NRP and NMP, as a superior alternative for mitigating IRI (148). The topic in relation to the role of ischemia and disturbed metabolism will be resumed below in Section 5.1.2.

#### 4.3.2 Regulated cell death and emission of DAMPs in DCD

As outlined above, types of RCD and the associated emission of DAMPs occur to a considerable extent in DBD donors and have been convincingly demonstrated. In contrast, the situation for DCD donors is less clear. On the one hand, expression of DAMPs (HSP70, peroxiredoxins) has experimentally been shown to be upregulated during the first 30 min of warm ischemia (149). However, on the other hand, since there is probably neither sufficient time nor energy available during fWIT in cDCD, it is reasonable to doubt that types of RCD develop and, consequently, DAMPs are released from these dying cells. But what about earlier harmful DAMP-inducing events experienced by uDCD donors,? Moreover, what about harmful DAMP-promoting events encountered by cDCD donors in the ICU before the process of dying was actively initiated? To gain a fuller understanding of the potential occurrence of RCD→DAMPs in DCD donors, these scenarios should also be considered.

##### 4.3.2.1 RCD-promoting cell death pathways in DCD

To our knowledge, there are no targeted studies on the role of RCD→DAMPs during prolonged warm ischemia times in DCD donors. On the other hand, there is some preliminary evidence suggesting that at least cell-death pathways may be activated during such ischemic conditions, which may later lead to RCD during cold storage.

Of interest in this context is a recent study on lung grafts from rats demonstrating that prolonged warm ischemia after induction of cardiocirculatory death-initiated, RIP kinase-mediated necroptosis, which was exacerbated by cold storage insult and enhanced lung graft injury (150). In support of this study is an analysis of tissue biopsies from human DBD and DCD donor lungs, revealing that DCD lungs display a transcriptome signature of pathways associated with cell death, apoptosis and necrosis (151). Such examples from the still limited research on RCD in DCD emphasize the consideration that the sources of DAMPs release in DCD must be sought in previous harmful episodes experienced by DCD donors.

It stands to reason that in an ICU, harmful situations can arise for patients with periodic hypoxemic/hypoxic conditions, which are known to trigger RCD types such as ferroptosis and necroptosis (152, 153). More essential, however, seem to be events such as continuous active mechanical ventilation and cardiac arrest followed by successful resuscitation, in which potential DCD donors may already develop RCD and DAMPs even before the final decision to initiate the DCD procedure is made.

##### 4.3.2.2 Ventilator-induced lung injury

Mechanical ventilation causes RCD! For example, studies on a mouse model of ventilator-induced lung injury (VILI)—encouraged by observations of higher RIPK3 levels in patients requiring ventilator support—provided first evidence suggesting that VILI may induce necroptosis (154). Moreover, in a more recent study on a mouse model of VILI, evidence from several findings was provided suggesting that ferroptosis occurs during the process of VILI (155). Consistent with these experimental observations are clinical studies on bronchoalveolar lavage fluid in patients under mechanical ventilation showing that levels of the DAMPs HMGB1, HSPs, and S100A9/S100A12 are elevated [cf (156).]. Plausibly it can be assumed that forms of RCD serve as the source of these DAMPs and, further, that they enter the circulation to reach potential donor organs [cf (157).].

##### 4.3.2.3 Cardiac arrest/successful resuscitation-induced ischemia/reperfusion injury

Similarly, as shown in experiments on a rat model of cardiac arrest with subsequent successful resuscitation, both pyroptosis and necroptosis are involved in the systemic inflammatory response (158). And in this context, one may also discuss that hypoxia, which may occur during cardiac arrest, may at least initiate pathways leading to ferroptosis later on (153).

These experimental aspects lead over into clinical studies: Cardiac arrest followed by successful resuscitation represents a harmful event to ICU patients: the life-threatening event of post-cardiac arrest syndrome, which is often associated with IRI (159). DAMPs are reportedly proposed to play a crucial role in the pathogenesis of this complication. For example, plasma levels of DAMPs including S100A12, HSP70, nDNA, and mtDNA were found to be elevated after cardiac arrest and out-of-hospital resuscitation (cf (156). Moreover, also in patients with post-cardiac arrest syndrome after out-of-hospital cardiac arrest, HMGB1 was detected and shown to be associated with neurological outcomes (160).

#### 4.3.3 Activation of the donor's innate immune system in donation after circulatory death

As outlined above, under DBD condition, there is a typical creation of a systemic autoinflammatory syndrome that reflects the substantial DAMP-promoted activation of cells of the donor's innate immune system. In DCD, data reported from this research area are still meager, although recent studies provided first evidence indicating that, at least in some cases and under certain conditions, the innate immune system of DCD donors is also activated, albeit apparently to a lesser extent than in DBD donors. For example, a prospective controlled multicenter trial on DBD and DCD lung donors conducted by Sandiumenge et al. (161) revealed that plasma levels of all measured cytokines were found to be numerically higher in DBD donors compared to DCD donors, with IL-6, IL-10 and IL-8 reaching statistical significance. These findings provide indirect but compelling evidence that DAMPs circulate and function in organs of DCD donors, thereby promoting—albeit more mild—autoinflammation. And it is reasonable to argue that these putative DAMPs are emitted during previous harmful events occurring in the ICU such as ongoing VILI and/or episodic cardiac arrest followed by successful resuscitation associated with induction of IRI. However, further targeted and systematic studies are needed to validate the emission of RCD-associated DAMPs, their role in triggering systemic autoinflammation, and, critically, their involvement in activating donor-derived DCs in DCD donors.

#### 4.3.4 Résumé: the immunogenicity of organs from DCD donors

Given the varying categories of DCD donors, it is not currently feasible to draw definitive conclusions regarding the impact of DCD-related harmful events on the activation of the peripheral innate immune system. Similarly, the role of DAMPs in activating PRR-bearing cells of the donor cannot be clearly defined, as systematic studies investigating their emission in the organs of DCD donors are lacking. Nevertheless, given the extensive understanding of the phenomenon that every injury induces DAMPs, it is reasonable to anticipate that DAMPs are contextually generated and emitted in DCD donors to cause systemic autoinflammation and promote activation of donor iDCs, which mediate direct allorecognition and confer immunogenicity in the recipient (Figure 7) (for further details, see Part 2). In the case of cDCD donors, this idea is supported by the abovementioned scenario of their treatment in the ICU. Thus, the therapeutically mediated process of VILI has been shown in studies to promote occurrence of RCD and associated emission of DAMPs. Similarly, cardiac arrest → successful resuscitation-evoked IRI is thought—similar to what is seen in post-cardiac arrest syndrome—to trigger emission of RCD→DAMPs.

At the end, this kind of an acquired immunogenicity of DCD organs—as discussed here- could even match that of DBD organs despite the different sources and obviously lower amount of DAMPs. Indeed, this aspect, as previously reviewed by us (64), may help explain the observation that renal allografts from DCD donors, despite a higher incidence of DGF of 73% compared to 27% in DBD donor kidneys, exhibit a similar rate of acute rejection episodes and show no significant differences in long-term outcome when compared to kidneys from DBD donors. Similarly, liver and lung allografts from DCD donors have also been shown to yield outcomes comparable to those from standard brain-dead donors. It would be useful to conduct further studies to validate the hypothetical background to these observations.

### 4.4 Donor organ preservation procedures

#### 4.4.1 Static cold storage preservation

Static cold storage (SCS) can still be regarded as the standard preservation method of organs from nonmarginal DBD donors, although it was shown to cause oxidative injury to cellular components (162). The procedure slows down cell metabolism and lowers the oxygen demand of the organ. However, cell metabolism does not fully cease; instead, it continues as anaerobic metabolism at a low rate, which leads to a depletion of ATP stores and accumulation of succinate that promotes the production of ROS (147, 163). However, after adoption of SCS for the preservation of organs from ECD donors including DCD donors, it turned out that the outcome of these transplant organs was inferior due to aggravated IRI (164, 165).

As a possible cause for inferior outcomes, first evidence was provided suggesting emergence of cell-death pathways associated with RCD (151). In support of this observation are studies on DCD porcine livers, showing a significant increase in the expression of necroptosis biomarker, pMLKL and the ferroptosis-associated biomarker GPX4 after 24 h of cold preservation (166). Similar evidence was reported from studies on rat liver grafts (167).

In addition, there is preliminary evidence for the emission of DAMPs during SCS. Thus, in studies on samples of organ preservation solution of explanted livers from deceased donors, collected after SCS, DAMPs such as HMGB1, HSP70, and free dsDNA were identified. In addition, several of these molecules were found to induce both priming and activation of the NLRP3 inflammasome in human myeloid cells (168). Notably, cold ischemia time and DCD donation was observed to negatively influence the DAMP signature.

In considering these results, it is arguable that at least some of the DAMPs may have been emitted earlier, accumulating under DBD or DCD conditions, or even earlier during previous damaging events. Therefore, further research on this significant topic is anticipated.

#### 4.4.2 Normothermic perfusion preservation

Normothermic perfusion preservation techniques were designed and developed—among others—to reduce ischemic damage due to the vasoconstrictive effects of cold graft washout with the SCS solution (169). On the other hand, the application of these techniques basically allows the continuation of processes related to occurrence of RCD and release of DAMPs. And indeed, there is growing evidence from experimental and clinical studies indicating that normothermic oxygenated machine perfusion of DCD transplant organs triggers the entire cascade of reperfusion injury as evidenced, for example, by the release of DAMPs (170–173)*.* Overall, however, a conclusive consideration of the risk of NMP-induced IRI in clinical transplantation of marginal organs from DCD and ECD donors is still pending. In particular, the risk of activation of donor-derived DCs contributing to direct allorecognition needs to be explored. The topic will be resumed and discussed in detail in Part 2, Section 3.2.2.

### 4.5 Conclusion

The scenarios outlined in this chapter impressively illustrate the periodic, injury-induced transformation of originally “native” donor organs into transplant organs that exhibit a high level of immunogenicity. Strikingly, this immunogenicity is already pronounced even before the allograft experiences IRI in the recipient. Mechanistically, the release of DAMPs from forms of RCD confers this type of immunogenicity, which, in the context of an autoinflammatory tissue environment, is evident in the activation of donor-derived DCs that mediate direct allorecognition after transplantation.

## 5 Ischemia/reperfusion injury to the donor organ in the recipient: the critical determinant of alloinflammation and transplant immunogenicity

The impact of IRI on acute and chronic allograft rejection events described in 1994 was the basis of formulating the danger/injury hypothesis (4). In three anecdotal articles subsequently published in the late 1990s, a spectrum of IRI-associated processes was addressed and discussed including the nature of ROS-mediated oxidative injury, the activation of an inflammatory response via secretion of cytokines (e.g., IL-1β, IL- 6, IL-8, TNF) and chemokines, complement activation, and upregulation of cell adhesion molecule expression. Particular focus was given to upregulation of MHC molecule and costimulatory molecule expression on DCs leading—along with alloantigen presentation and via direct and indirect allorecognition—to full T cell alloactivation of recipient T cells (174–176). In a following article (6), preliminary evidence was presented that IRI to allografts promotes the generation of DAMPs such as HSPs that trigger activation of donor- and recipient-derived TLR-bearing DCs. Once developed into immunostimulatory APCs, activated DCs stimulate subsequent activation of T and B cells of the recipient's adaptive alloimmune system, leading to acute allograft rejection.

**Figure 8:**
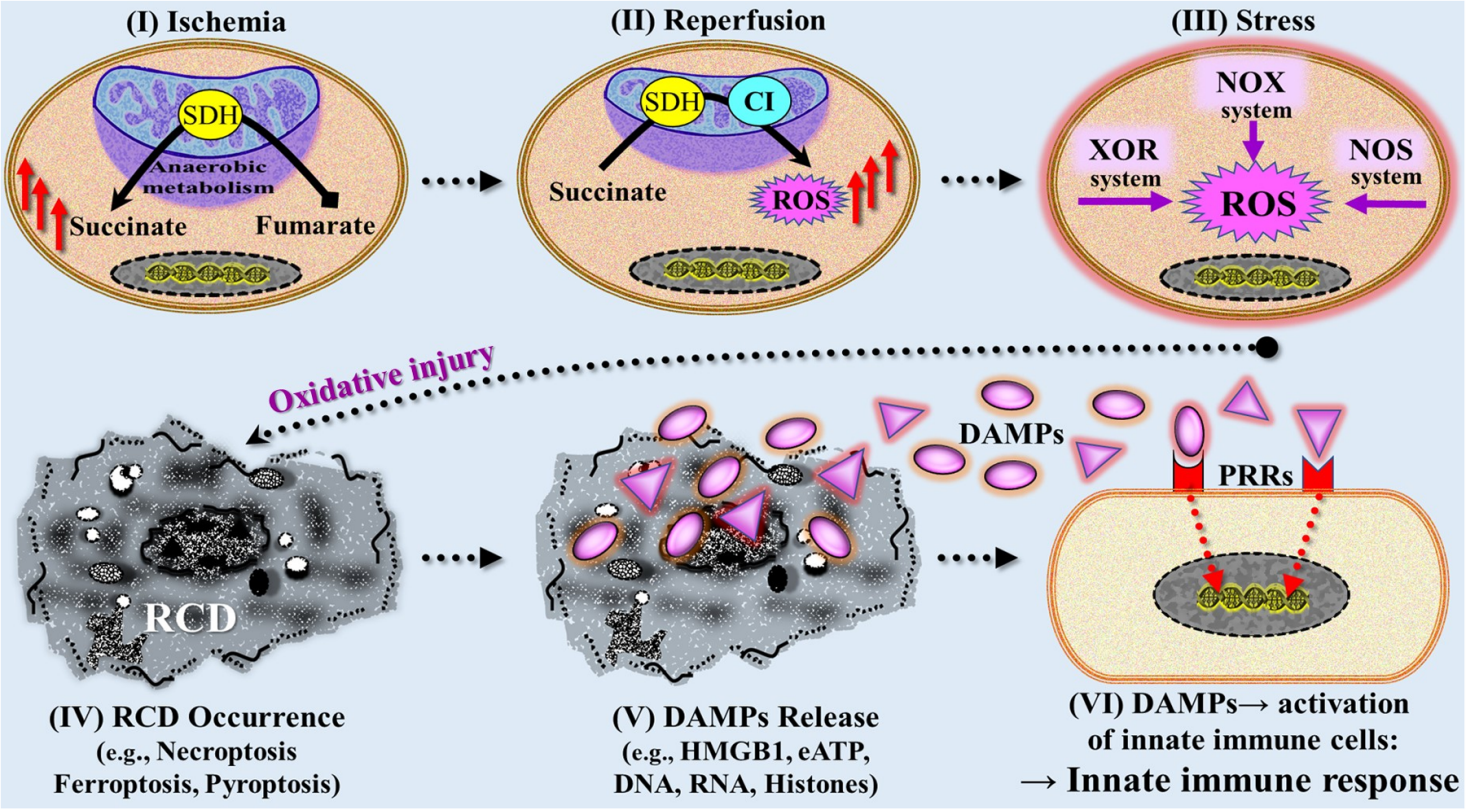
Simplified and rough schematic diagram illustrating a model with six stages mechanistically involved in ischemia reperfusion injury-induced innate immune responses. **(I)** During initial (warm) ischemic tissue condition, the succinate dehydrogenase (SDH) operates in reverse, reducing fumarate to succinate that accumulates within mitochondria. (II) On subsequent reperfusion, the accumulated succinate is rapidly oxidized by SDH causing generation of reactive oxygen species (ROS) as a result of reverse electron transport through mitochondrial complex I (CI). (III) Following initial reperfusion, other ROS-producing mechanisms contribute to increased generation of ROS including the extramitochondrial xanthine oxidasoreductase (XOR) system, NADPH oxidase (NOX) system, and nitric oxide synthase (NOS) system. (IV) Severe ROS-mediated oxidative injury leads to various types of regulated cell death (RCD) such as ferroptosis, necroptosis, and pyroptosis, which are associated with **(V)** release of large amounts of DAMPs (compare Figure 6). (VI) DAMPs interact with pattern recognition receptors (PRRs) on/in multiple cells of the innate immune system, thereby triggering an innate immune response (compare Figures 4, 5). eATP, extracellular ATP; HMGB1, high mobility group protein B1.

Since the introduction of innate immunity as a concept involved in danger signal/TLR-driven innate immune pathways leading to alloimmunity → allograft rejection (1), numerous excellent review articles on mechanisms of IRI have been published, building upon and expanding this earlier work, with the mechanisms now understood with focus on the innate immune response (164, 171, 177–185). Accordingly, the current conceptual model of a mechanistic/pathogenetic axis from initial ischemia to IRI-induced innate immune responses can be roughly divided into six consecutive stages: (1) ischemia (hypoxia/hypoperfusion) → (2) reperfusion-induced, ROS-caused oxidative injury → (3) extended (oxidative) stress responses → (4) emergence of RCD types → (5) emission of DAMPs → (6) DAMP-triggered activation of PRR-bearing cells of the innate immune system (e.g., neutrophils, macrophages, DCs, endothelial cells) (Figure 8). In the following, these new insights into mechanisms of IRI are presented in a concise telegram style.

### 5.1 Ischemia → reperfusion → oxidative injury

The phases of ischemia a transplant organ is exposed to are different for DBD and DCD. In traditional conventional recovery of organs from brain-dead donors, ischemia is defined as first warm ischema time during removal, followed by a long cold ischemia time in standard SCS and a second warm ischemia time during implantation of the allograft in the recipient. Assessment and description of the degree of IRI to an allograft over the past fifty years has been standardized in relation to this conventional organ recovery/preservation protocol applied in DBD donors.

Compared to DBD, DCD presents a unique set of challenges due to the injury brought about by prolonged warm ischemia times. This detrimental extended ischemic period is reportedly linked to the generally observed aggravation of IRI to the donor organ, which is often associated with complications commonly observed with DCD organs in recipients.

Obviously, the severity of IRI is strongly influenced by the duration of warm ischemia times, requiring a closer examination of the molecular processes that take place during ischemia to promote initial oxidative injury during subsequent reperfusion.

#### 5.1.1 Dominant role of reactive oxygen species in ischemia reperfusion injury

While a variety of molecular mechanisms have been proposed to explain the complex phenomenon of IRI, the excessive production of ROS that overrules the autonomous cell's antioxidative defense system continues to receive—in tandem with lipid peroxidation and increase in intracellular iron concentration—most attention as a critical factor in the genesis of this kind of oxidative injury. In earlier studies, it was already found that the initial injurious event upon reperfusion is a burst of ROS production from mitochondria (186, 187). Moreover, it could be demonstrated that mitochondrial ROS not only induce that type of an acute injury but also instigate the pathophysiological processes that develop subsequently over days and weeks following reperfusion (188). Today, the notions still hold that it is this production of large amounts ROS during the anoxia → hypoxia → reoxygenation phase that is deleterious over a prolonged period, as it promotes innate immune proinflammatory pathways and triggers cell-death- trajectories.

This concept of ROS-induced injury has found a way into the world of transplantation: Since the time of publication of the first clinical trial (4), production of ROS during IRI has been confirmed in transplant models and human transplant organs (171, 189–192). However, the mechanisms involved in the production of ROS have been the subject of constant discussion over the past decades. Recently, however, new research findings have directed attention to ischemia-related metabolic disorders.

#### 5.1.2 Metabolic processes promoting the initial generation of reactive oxygen species

Following abrupt cessation of blood flow, cells are confronted with substantial oxygen deprivation. Cellular hypoxia leads to a disruption of the electron transport chain in mitochondria resulting in the progressive decline of the concentrations of adenine nucleotides and nucleosides (adenosine, inosine). Depletion of mitochondrial ATP, in the absence of oxygen, results in a switch from cellular respiration to anaerobic cellular metabolism. In addition, decreasing mitochondrial ATP production leads to depletion of metabolic substrates and accumulation of detrimental metabolites and ions, including Na^+^, K^+^, and Ca^++^, due to the failure of ATP-dependent ion-exchange channels. This leads to intracellular acidosis and edema (cell swelling) and impaired enzymatic activity in the cytoplasm (184, 193–196).

In relation to these metabolic molecular disorders, several mechanisms have been described for warm ischemia times to promote the generation of ROS during subsequent reperfusion. Most attention has recently been paid to studies in mice showing that, during ischemia, succinate accumulates within mitochondria, which, on subsequent reperfusion, is rapidly oxidized by succinate dehydrogenase at the mitochondrial complex II causing generation of ROS as a result of reverse electron transport through mitochondrial complex I (147, 197, 198). In fact, it is the succinate-fueled reverse electron transport that is now firmly believed to provide the initial burst of ROS that leads on to IRI injury [although this could not be confirmed in clinical studies in the context of kidney transplantation (199)].

#### 5.1.3 Oxidative stress-mediated pathologies

After the reperfusion stage, when oxygen delivery is restored through blood flow, additional ROS-producing mechanisms beyond succinate accumulation become active, contributing to heightened generation of ROS. This increase in ROS is facilitated by an anaerobic metabolism-dependent lower concentration of antioxidative agents in ischemic cells as shown for the cellular antioxidant glutathione [discussed in (200)]. Among these other potential sources of ROS, the extramitochondrial xanthine oxidase system, NADPH oxidase (NOX) system, and nitric oxide synthase system—preferentially located in vascular cells—have emerged as the most likely contributors to reperfusion-induced oxidative stress (201) (Figure 8). As reviewed by Granger and Kvietys (201), xanthine oxidase system drives ROS generation by oxidizing hypoxanthine to xanthine and xanthine to uric acid, whereas the NOX enzymes generate superoxide and hydrogen peroxide via the activation of *hypoxia-inducing factor* (HIF)-1*α*, phospholipase A2, TNF-α, IL-1β, IFN-*γ*, and angiotension II.

Of note, activation of these extramitochondrial ROS-producing enzyme systems appears to occur after the initial burst of mitochondrial ROS. The ROS generated by these enzymes are thought to drive oxidative stress-mediated processes over the next hours and days. Such processes include direct oxidation of macromolecules—such as membrane lipids, structural proteins, enzymes, and NAs—as well as the activation of stress responses like ER stress (202). Due to excessive production of ROS, these cellular stress responses fail to repair molecular damage, prompting the cells to undergo RCD.

### 5.2 Regulated cell death

Some types of RCD discussed above in Section 3.2 are also implicated in IRI (Figure 8). Numerous studies on nontransplant and transplant IRI models have provided compelling evidence for prolonged and severe ischemia followed by reperfusion to induce various types of RCD of parenchymal and endothelial cells [reviewed in (185, 203)]. Of note, oxidative stress is involved in various types of RCD such as ferroptosis, necroptosis, pyroptosis, autophagy-dependent death, parthanatos, and NETosis [reviewed in (66)]. Here, just some selected aspects on ferroptosis, necroptosis, and pyroptosis will be discussed.

#### 5.2.1 Ferroptosis

Ferroptosis has been investigated in all types of organ IRI (204). In addition, as reviewed (185, 205), both preclinical and clinical studies have provided compelling evidence that ferroptosis also significantly contributes to cell death in IRI during organ transplantation. As a result from all these studies, ferroptosis is currently considered the key factor that leads pathogenetically to IRI; even more, it is believed that ferroptosis is the true cause of reperfusion injury (72).

As the main mechanisms govering the formation of ferroptosis in postischemic reperfusion settings, reperfusion-related excessive ROS accompanied by lipid peroxidation along with phospholipid oxidation products and an increase in intracellular iron concentration are being discussed (206) (compare Figure 6).

#### 5.2.2 Necroptosis

As early as 2013, necroptosis has already been described as a crucial element of IRI and proposed to promote—via emission of DAMPs—alloimmunity (82). It is now well-established that necroptosis plays a critical role in the pathogenesis of IRI across various organs, including kidney (207), liver (208), and the heart (209). Moreover, necroptosis has also been confirmed to drive DAMP –triggered trajectories leading to allograft rejection [for reviews, see (210, 211)]. Interestingly, in a time course analysis on a mouse model of unilateral warm kidney IRI, the investigators identified the period 3–12 h after reperfusion as a critical phase for the activation of necroptosis in proximal tubular cells (212). Additonally, after 12 h, the predominant pattern of pMLKL staining was found to shift from cytoplasmic to membrane, signifying the transition to the terminal phase of necroptotic cell death.

#### 5.2.3 Pyroptosis

Studies on various models of IRI such as myocardial IRI have provided evidence suggesting that pyroptosis contributes also to the pathogenesis of IRI. Its development during such oxidative injury is differently discussed, for example, as the result of the priming and triggering of the NLRP3 inflammasome by locally released DAMPs or a consequence of ROS (213, 214). Interestingly, in the context of liver transplantation, damaged cells have been shown to release various ROS and DAMPs during SCS, which trigger activation of the NLRP3 inflammasome and lead to pyroptosis upon reperfusion (215). As discussed by the authors, this process may influence the inflammatory response during the early phase of acute rejection in liver transplantation.

### 5.3 Emission of DAMPs

A model of IRI-associated DAMPs in promoting innate alloimmune responses leading to allograft rejection was first published as early as 2002/2003 (1, 6). Over the past decades, many reports on involvement of DAMPs in nontransplant and transplant models of IRI have been published and confirmed these earlier observation. In fact, previous reports have already noted that most of the *in vivo* evidence for the existence of DAMPs operating as TLR agonists is derived from studies on IRI (216). Indeed, there is now general agreement from studies in nontransplant and transplant models that DAMPs promote innate immune processes caused by IRI. A few selected aspects are presented here.

#### 5.3.1 Experimental nontransplant models

The generation and emission of DAMPs has been demonstrated in numerous nontransplant IRI models. For example, in myocardial IRI, well-recognized DAMPs such as HMGB1, extracellular DNA and histones have been identified (217–219). Similarly, in cerebral IRI, DAMPs such as HMGB1, mtDNA, eATP as well as iDAMPs such as IL-1β have been described to promote innate immune responses (220). In kidney IRI, DAMPs such as HMGB1, mtDNA, and peroxiredoxin 1 (Prdx1) have been demonstrated (221–223). Similarly, as reviewed elsewhere (224), serum and tissue levels of the DAMP eCIRP were also elevated in a number of organ-targeted ischemia and reperfusion models characterized by sterile inflammation, including rodent models of hepatic ischemia, mesenteric ischemia, ischemic acute kidney injury (AKI), and stroke. Such examples could be continued at will, but due to space constraints, they will not be elaborated upon here.

#### 5.3.2 Transplant patients and experimental transplant models

As reviewed by us (8), there is a growing number of reports on the role of DAMPs in driving innate alloimmune responses resulting in allograft rejection. For example, in liver IRI, HMGB1, histone/DNA complex, and eATP as well as iDAMPs such as TNF and INF-I have been identified as best characterized DAMPs, which activate TLR4, TLR9 and the NLRP3 inflammasome (225). Studies on renal IRI, including renal transplant IRI, have shown that necrotic supernatant derived from human renal tubular epithelial cells contains molecules that function as DAMPs triggering TLR and NLR signaling pathways. This leads to the production of proinflammatory mediators and promotes the proliferation of renal tubular epithelial cells (226). As reported from other studies (227), mtDNA has been identified as a key DAMP involved in IRI, accelerating the progression of IRI by inducing inflammation and IFN-I responses. In this context, the authors argued that mtDNA could serve as a potential biomarker for predicting post-transplant renal allograft function. Similarly, studies on cardiac allograft IRI identified key DAMPs, such as HMGB1, HSP70, mtDNA, and eATP, which interact with their respective PRRs (217). Based on these findings, the researchers concluded that this upstream interaction plays a critical role in establishing a proinflammatory milieu, which significantly contributes to the harmful effects of IRI in heart transplantation. Likewise, as noted in a review with reference to pulmonary transplantation (228), IRI to lungs is driven by sterile inflammation, where DAMPs released from dying cells are recognized by PRRs.

#### 5.3.3 Post-translational modifications in controlling DAMP-triggered innate immune responses

While the role of IRI in generating RCD→DAMPs is increasingly well-documented, its impact on epigenetic regulation of DAMP-triggered, PRR-mediated innate immune responses—especially through post-translational modifications (PTMs) like oxidative PTMs -remains largely overlooked and underexplored (229). A compelling example that underscores the profound impact of IRI-induced PTMs in controlling the function of DAMPs refers to the prototypic DAMP, HMGB1.

In fact, the redox state of HMGB, achieved by oxidation-driven extensive PTM, is essential for its active secretion from the nucleus, as it promotes cytoplasmic accumulation and extracellular release of this nuclear DAMP during cell stress (230, 231). In addition, the redox state of HMGB1 dictates its binding to different PRRs, ultimately shaping its dual role in driving proinflammatory or counterbalancing anti-inflammatory, inflammation-resolving, and immunosuppressive responses (230, 231). For example, in its function as a redox state-depending chemoattractant, HMGB1 can drive immune cell migration, either mobilizing proinflammatory leukocytes (232) or, depending on the context, recruiting immunosuppressive regulatory T cells (233, 234). Likewise, the redox state of other DAMPs such as S100 proteins has been shown to regulate their function by shifting their pro-inflammatory activity towards protective anti-inflammatory effects (235).

In sum, while considerable progress has been made in understanding how PTMs regulate innate immune responses, further targeted research is essential to unravel their critical role in IRI-induced generation and emission of DAMPs—especially in deciphering their context-dependent proinflammatory or inflammation-resolving, immunosuppressive functions.

### 5.4 Activation of the donor's and recipient's innate immune system

#### 5.4.1 Transplant alloinflammation

During reperfusion of the donor organ in the recipient, IRI-induced RCD→DAMPs activate PRR-bearing innate immune cells, including resident donor cells such as endothelial cells, epithelial cells, and iDCs as well as immigrated mobile cells of the recipient such as neutrophils, macrophages and iDCs (Figure 8). In turn, these cells secrete various inflammatory mediators, including cytokines such as TNF, IL-1β, and IFN-I,which operate as iDAMPs, as well as IL-6 and IL-8 and chemokines such as CXCL1, CXCL2, and CXCL5. Additionally, the complement cascade is activated leading to the formation of the soluble bioactive peptides, C3a and C5a (acting as iDAMPs), and the membrane attack complex, which results in the recruitment of inflammatory cells (228, 236–241).

In view of the fact that an organ is transplanted from a donor into a recipient, a semanic pecularity of the term inflammation can be noted: The originally “autoinflamed” organ of the donor can now be referred to as an “ alloinflamed” organ in the recipient.

#### 5.4.2 Activation of donor-derived and recipient-derived dendritic cells

There is a growing body of compelling evidence from studies in various experimental settings, particularly in tumor setups, that DAMPs are able to promote maturation of iDCs into immunostimulatory DCs (49, 61, 242–246). In organ transplantation, DAMP-driven, PRR-mediated maturation of residual resident donor iDCs and graft-infiltrating (alloantigen engulfing and processing) recipient iDCs into immunostimulatory DCs is considered the key events in oxidative injury-activated innate immunity, which triggers adaptive alloimmunity (172, 179, 180, 183, 247, 248).

Notably, this maturation process enables the DCs to present and cross-present antigenic peptides to naïve CD4^+^ T cells and CD8^+^ T cells, which generally serve as critical events in initiating many adaptive immune responses. The innate alloimmune response that culminates in allograft rejection is a distinct immunological variant, where the priming of recipient alloreactive T cells with alloantigens is executed by both donor-derived and recipient-derived immunogenic DCs through direct, indirect, and semi-direct pathway of allorecognition (172, 249–251) (the topic will be resumed in Part 2, Section 2.1).

Of note, this core process of injury-induced, DAMP-driven, PRR-mediated maturation of DCs in peripheral tissues is marked by a remarkable phenotypic metamorphosis of these professional APCs. These extensive changes involve the upregulation of MHC II molecules (signal 1), upregulation of costimulatory molecules, for example, B7-1 (CD80) and B7-2 (CD86), ICOS-L, CD40, and OX40l (signal 2), and secretion of T cell-polarizing cytokines (signal 3), all of which are required to fully activate naïve T cells. Additionally, migratory molecules such as C-C chemokine receptor type 1 (CCR1), CCR2, CCR5, and CCR7 enable DCs to migrate from the periphery to the host's secondary lymphoid organs where they present processed peptidic antigens in the frame of MHC molecules to naive T cells. The result is the mounting of a specific “tailor-made” adaptive immune response [for further reading, see (172, 252–257)].

#### 5.4.3 DAMPs involved in activation of dendritic cells

Remarkably, there is evidence for a collaboration of various DAMPs in the activation of iDCs to mature immunostimulatory DCs (57, 64, 172). Here, we will mainly focus on the DAMPs released from types of RCD, that is, cDAMPs as well as distinct iDAMPs, which are secreted by cDAMP-activated innate immune cells.

For example, the IRI-induced DAMP HMGB1 has been shown to promote DC maturation and thereby Th1 cell polarization. According to data from several studies (53, 258–260), HMGB1, mainly via recognition by its cognate receptor RAGE, can be considered a potent immunostimulatory DAMP that is able to promote DC-mediated cross-priming leading to subsequent T cell-mediated adaptive immunity. Similar to HMGB1 and as shown in other sets of studies**,** members of the HSP70 family promote—via binding to TLR2 and TLR4—the maturation of immunostimulatory DCs, which are then able to elicit Th1 responses (261, 262). Extracellular ATP has also been demonstrated to contribute to DC maturation (263). In addition, components of the damaged ECM released during injuries like IRI, such a hyaluronan (264) and heparan sulfate (265), have been observed to promote DC maturation through TLR4 activation.

And not to forget here self NAs: indeed, though not shown in IRI settings, there is indirect evidence suggesting that endogenous NAs can promote maturation of iDCs. For instance, DNA and RNA have been shown to activate DCs through TLR7 and TLR9 (266) or cGAS→ STING (267), often acting in complex with chaperones like HSPs and HMGB1 (242). Further indirect support for the notion that IRI-associated release of NAs may also contribute to DC maturation stems from studies showing that NA-sensing receptors such as TLR3 and TLR9 promote DC maturation (268, 269). In this context, a recent study on human neutrophils is interesting showing that mtDNA and chromatin DNA contained in NETs can promote maturation of DCs (270).

Additionally, certain iDAMPs known to be implicated in IRI have been demonstrated to activate immunostimulatory DCs. They include type I IFNs (271, 272), TNF (273), and members of the IL-1 familiy such as IL-1β (274). A scenario that impressively illustrates involvement of both iDAMPs and cDAMPs in activation of DCs refers to research on ICD in tumor immunology: cDAMPs like HMGB1, HSPs, eATP, and CALR, as well as iDAMPs such as IFN-I are essential DAMPs required to activate DCs to initiate effective antitumor immune responses (275–277).

### 5.5 Conclusion

Ischemia/reperfusion injury of tissues involves a highly intricate network of interconnected pathophysiological events and signaling pathways. Recent advances in research on RCD and DAMPs have now made it possible to trace a six-step arc from initial tissue ischemia via the induction of RCD types to DAMP-triggered activation of PRR-bearing cells of the innate immune system (Figure 8).

In organ transplantation, this IRI-induced scenario includes the DAMP-driven activation of PRR-bearing donor-derived and recipient-derived DCs that confer the final peak immunogenicity of the donor organ: Via processes of direct, indirect and semi-direct allorecognition after migration to the secondary lymphoid organs of the recipient, these DCs—activated in the context of a detrimental alloinflammatory environment—initiate and amplify an adaptive T cell-/B cell-mediated alloimmune response. Notably, these DAMP-orchestrated alloinflammatory and allo-immunogenic processes are obviously more pronounced when organs from DCD donors are used, as the primary IRI during initial perfusion is exacerbated by their prolonged exposure to warm ischemia times (compare stage (I) Ischemia in Figure 8) (278, 279).

In sum, in analogy to a “viral load”, one could insinuate: it is the “DAMPs load” that determines the degree of IRI-associated allograft dysfunction and the intensity of immunogenicity in initiating an alloimmune response.
